# Targeted Sequencing Reveals Large-Scale Sequence Polymorphism in Maize Candidate Genes for Biomass Production and Composition

**DOI:** 10.1371/journal.pone.0132120

**Published:** 2015-07-07

**Authors:** Moses M. Muraya, Thomas Schmutzer, Chris Ulpinnis, Uwe Scholz, Thomas Altmann

**Affiliations:** 1 Leibniz Institute of Plant Genetics and Crop Plant Research (IPK) Gatersleben, Corrensstraße 3, D-06466, Stadt Seeland, Germany; 2 Department of Plant Science, Chuka University, P.O. Box, 109–60400, Chuka, Kenya; University of Guelph, CANADA

## Abstract

A major goal of maize genomic research is to identify sequence polymorphisms responsible for phenotypic variation in traits of economic importance. Large-scale detection of sequence variation is critical for linking genes, or genomic regions, to phenotypes. However, due to its size and complexity, it remains expensive to generate whole genome sequences of sufficient coverage for divergent maize lines, even with access to next generation sequencing (NGS) technology. Because methods involving reduction of genome complexity, such as genotyping-by-sequencing (GBS), assess only a limited fraction of sequence variation, targeted sequencing of selected genomic loci offers an attractive alternative. We therefore designed a sequence capture assay to target 29 Mb genomic regions and surveyed a total of 4,648 genes possibly affecting biomass production in 21 diverse inbred maize lines (7 flints, 14 dents). Captured and enriched genomic DNA was sequenced using the 454 NGS platform to 19.6-fold average depth coverage, and a broad evaluation of read alignment and variant calling methods was performed to select optimal procedures for variant discovery. Sequence alignment with the B73 reference and *de novo* assembly identified 383,145 putative single nucleotide polymorphisms (SNPs), of which 42,685 were non-synonymous alterations and 7,139 caused frameshifts. Presence/absence variation (PAV) of genes was also detected. We found that substantial sequence variation exists among genomic regions targeted in this study, which was particularly evident within coding regions. This diversification has the potential to broaden functional diversity and generate phenotypic variation that may lead to new adaptations and the modification of important agronomic traits. Further, annotated SNPs identified here will serve as useful genetic tools and as candidates in searches for phenotype-altering DNA variation. In summary, we demonstrated that sequencing of captured DNA is a powerful approach for variant discovery in maize genes.

## Introduction

Uncovering genotype-phenotype associations is one of the central goals in a path towards plant improvement, and this requires the accurate detection of different types of genomic variation. The *Zea mays* subsp. *mays*, commonly referred to as maize, reference genome sequence [1] provides a major foundation for maize molecular genetics. However, accurately linking genomic variation to the expression of certain traits requires the systematic investigation and knowledge of the entire spectrum of DNA sequence diversity, including single nucleotide polymorphisms (SNPs), insertions/deletions (INDELs), copy number variation (CNV), and presence/absence variation (PAV), as well as the frequency with which they occur in certain populations. Substantial progress has been made towards this goal in maize, and the insight gleaned from the sequence and structural variation identified in this organism [2–4] has expanded our knowledge of maize evolution and biology and stimulated genome research. Furthermore, the use of known large-scale sequence variation information to develop a comprehensive (50k) SNP genotyping array [5] has demonstrated usefulness in genome-wide association studies (GWAS) [6].

Maize, one of the most important crops for human food and livestock feeds, has a large and complex 2.365 Gbp genome, only 7.5% of which is predicted to encode genes [7]. Maize exhibits very high levels of both phenotypic and genetic variation, with SNP frequencies among maize inbreds higher than those found between humans and chimpanzees [8]. This high level of genetic variation in maize is also manifested in its large pan-genome [9]. Substantial gains in traits of interest have been made through the selection of individuals for breeding based on their phenotypes, or their pedigree. More recently, genomics technologies, such as SNP typing, have been used to select individuals based on their genetic makeup. Targeted SNP genotyping technology has also enabled successful GWAS in maize [5,6,10–14]. However, this has known disadvantages, including ascertainment bias, since the SNPs used in these studies were chosen to exceed a minimum frequency of the rare allele, and SNP markers were selected from a limited number of divergent sources. Thus, the identification of very rare causal mutations might be complicated due to the failure of disequilibrium detection between casual mutations and typed SNPs [15,16]. In contrast, targeted sequencing of individual genomes would be expected to alleviate the polymorphism ascertainment bias. This would support the detection of rare functional variants and allow the discovery of complete haplotypes of genes, as well as CNV and PAV, both of which have recently been identified as an important sources of genomic variation [2–4], when a high coverage depth is attained. With the ever decreasing costs of sequencing and the advances in sequence capture technologies, approaches have been developed to catalogue genomic sequences, CNV, PAV, and rare variants (as opposed to earlier methods that were biased towards common variants) in maize. These can provide key resources for breeding initiatives aimed at mitigating the challenges of increased global demand for food, feed, fiber, and fuel.

As a step towards deciphering the genetic basis underlying important trait variation in maize, genes that are known or assumed to be involved in various aspects of plant growth, biomass production, or composition can be captured and sequenced in a wide panel of diverse maize inbred lines. This will yield a large inventory of genomic variation, which can be used to link genes, or genomic regions, with corresponding phenotypes. To this end, readily available sequence capture and next generation sequencing (NGS) technologies can be used. Unlike the whole genome sequencing that permits deep sequence coverage for only a small number of individuals, sequencing of captured target sequences will result in an enhanced proportion of sequence reads originating from regions of interest. Enrichment procedures allow the redistribution of sequencing efforts from whole genomes of a small number of genotypes to a restricted genomic fraction of a larger number of genotypes. The use of the latter approach, coupled with the available maize reference genome sequence [1], provides for a substantial contribution to the in-depth characterization of the genetic variation present in this organism.

In this study, we used sequence capture and 454 pyrosequencing to sequence the DNA of 21 maize inbred lines at 4,648 genes of interest and obtained, on average, a 19.6-fold sequencing depth in our raw data. High quality reads were aligned to the maize B73 reference genome sequence, and additionally assembled *de novo* to account for target genomic regions that are absent from the reference sequence. In order to detect the most comprehensive set of reliable SNPs in our maize collection and to determine the optimal variation calling method for our data, a wide range of read alignment and SNP calling tools were evaluated. Optimization of variant calling has previously been attempted by either finding the best alignment tool or by evaluating variant callers [17]. Here, we combined both approaches in order to obtain a broader perspective, and to evaluate how the read alignment procedure impacts the variant calling approach. We thus extended the collection of tools used in recent comparisons [18–20] and further included open source tools, as well as commercial SNP caller. The optimal SNP set was then used to investigate: (1) the level of sequence polymorphism in captured genes across the 21 maize inbreds, (2) the pattern of SNP distribution among the inbred lines and their functional annotation relative to B73, and (3) the pattern of gene variation and presence/absence genes in the studied inbred lines.

## Results

### Array design, sequence capture optimization, and assessment of capture efficiency

A high-density (2.1 million) oligonucleotide sequence capture microarray was designed using the B73 genome sequences. Captured target regions were selected ranging in sizes from 58–4,240 bp, with an average probe length of 75 bp. Probe selection settings allowed for up to 5-matches when aligned to the B73 RefGen_v1, and these probes were classified based on repetitiveness and locations relative to predicted genes (see Materials and Methods).

To modify and optimize available sequence capture protocols, a set of four customized qPCR loci was employed to estimate relative enrichment and to determine whether a capture was successful prior to sequencing (see Materials and Methods). We first tested capture efficiency of B73 DNA, and obtained high enrichments for all four control loci, ranging from 643- to 990-fold, with a mean of 749-fold (S1 Table). The enrichment of captured DNA from the remaining 20 inbred lines was then analyzed using the same control loci, and a high enrichment was found for all captured DNAs, ranging from 623- to 755-fold mean enrichment. NimbleGen recommends at least a 300-fold enrichment before committing sample libraries to the expensive and/or time-consuming downstream applications. In order to enhance the robustness and reliability of our DNA library quantification, we developed a method based on qPCR. Because our capture libraries, on average, contain 700 bp fragments, we used a plasmid fragment that results in a 725 bp PCR product when ligated to two 454 adaptors. The resulting PCR product, being of known quantity, was used as a standard for quantification of the captured DNA libraries. This leads to a more precise quantification of enriched captured DNA and minimizes the variation in cluster density or template-to-bead ratio, thus reducing the failure of GS FLX Titanium emulsion PCR (emPCR) preparation and subsequent sequencing. For example, using the standard DNA quantification protocol, the captured 454 sequence output for B106 and Mo17 was 50 and 37 Mbp, respectively. Whereas, after utilizing the optimized captured DNA quantification protocol, a sequence output of 235 Mbp and 312 Mbp, respectively, for B106 and Mo17 was achieved.

### 454 pyrosequencing

A total of 17,766,241 sequence reads, with an average read length of 363 bp, was generated, yielding more than 6.4 Gbp of sequence data (Table 1). The resulting average sequencing depth was estimated at 19.6-fold for the target regions. Among these reads, 10.6 million (60.0%) passed the quality trimming process and attained an average read length of 286 bp (78.8%). Upon quality trimming, the sequence depth declined to 9.3-fold (on average), which is still suitable for SNP detection (Table 1).

**Table 1:** Statistics of raw and preprocessed sequence data. A total volume of >6.4 Gbp of raw data was sequenced. Each of the 21 maize genotypes is designated by ‘D’ (Dent) or ‘F’ (Flint), according to which gene pool the line belongs.

Genotype	Total bases (Mbp)	Mean length (bp)	Raw reads	Quality trimmed reads	%	Base pairs used after trimming	%	Estimated coverage	Estimated coverage (trimmed)
B73 [D]	399	402	992,806	595,595	59.99	186,036,442	46.62	25.6	11.9
F353 [D]	200	397	503,938	324,246	64.34	101,734,580	50.82	12.8	6.5
P068 [D]	251	387	647,422	427,578	66.04	136,393,311	54.37	16.1	8.7
UH007 [F]	445	392	1,134,431	708,594	62.46	220,687,755	49.61	28.5	14.1
B101 [D]	230	294	782,915	433,769	55.40	95,362,651	41.47	14.7	6.1
B102 [D]	283	344	823,149	474,658	57.66	127,937,630	45.20	18.1	8.2
P107 [D]	273	340	804,167	433,932	53.96	112,451,816	41.12	17.5	7.2
B106 [D]	285	319	1,017,225	574,263	56.45	122,507,030	43.00	18.3	7.9
B111 [D]	342	382	895,724	631,430	70.49	195,512,634	57.16	21.9	12.5
F7059 [D]	303	371	817,130	504,308	61.72	147,282,945	48.64	19.4	9.4
Mo17 [D]	349	324	1,076,211	651,146	60.50	164,714,385	47.22	22.4	10.6
Mo24W [D]	308	382	804,525	450,652	56.01	133,649,118	43.43	19.7	8.6
NC358 [D]	340	387	879,208	537,185	61.10	164,251,568	48.31	21.8	10.5
P128 [D]	331	386	855,009	466,105	54.51	139,512,583	42.17	21.2	8.9
DK105 [F]	326	361	902,739	532,245	58.96	149,157,334	45.80	20.9	9.6
EA1070 [F]	337	370	909,460	532,589	58.56	155,772,546	46.26	21.6	10.0
EP1 [F]	343	380	903,222	486,639	53.88	146,530,019	42.68	22.0	9.4
F2 [F]	312	382	816,728	437,189	53.53	130,277,657	41.76	20.0	8.4
F7 [F]	316	387	816,142	515,399	63.15	166,516,436	52.76	20.2	10.7
Lo11 [F]	310	388	799,324	511,863	64.04	164,539,579	53.01	19.9	10.5
PH207 [D]	149	254	584,766	393,234	67.25	80,728,746	54.35	9.5	5.2
sum	6.432		17,766,241	10,622,619		3,041,556,765		412.2	195.0
avg.	306	363	846,011	505,839	60.00	144,836,036	47.41	19.63	9.28

### Evaluation of alignment methods and mapping results

**Fig 1:**
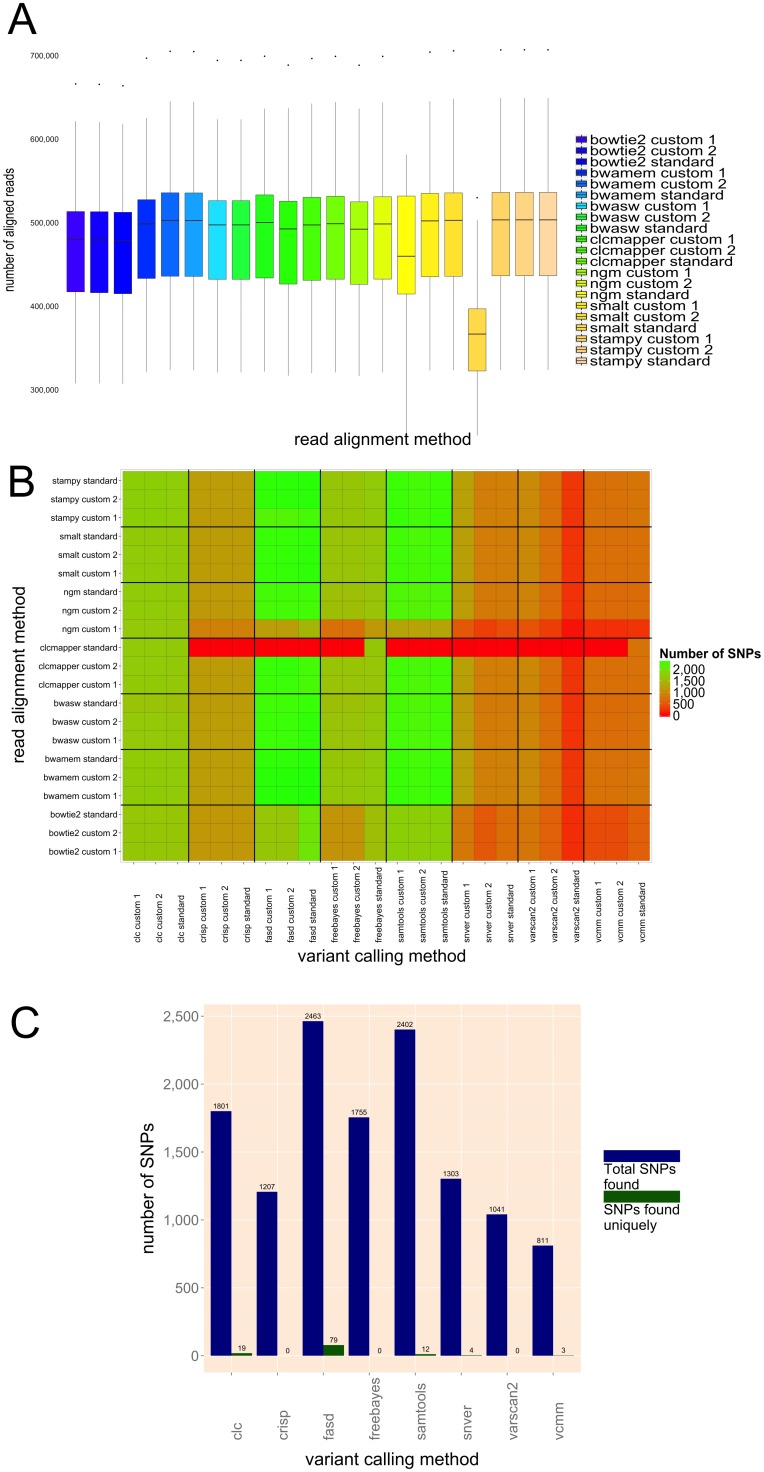
Evaluation of read alignment and variant calling methods. **(A)** Comprehensive illustration of 441 evaluated read alignment results. Each method is referenced in standard, and in two additional, parameter settings. The plots show the number of aligned reads, where the range for each bar illustrates the observed variability when different lines were used. **(B)** Heat map depicts the true positive sites in the 50k array. A total of 504 combinations of read alignment and variant calling methods were evaluated to identify recommended or less optimal applications (genotype NC358). **(C)** Variant caller performance compared to the 50k array. The total number of identified SNPs, as well as the number of unique SNPs, is depicted for each of the eight evaluated methods (genotype NC358).

The combined set of reads was aligned to the B73 reference genome (AGV3) using seven different read alignment tools (Table 2, see Materials and Methods), and the total fraction of mapped quality trimmed reads ranged from 95.66% (Bowtie2) to 99.81% (Stampy). On average 98.7% of the reads were aligned to the B73 reference. On average, 41.32% of the quality trimmed reads were mapped to the target sequence, ranging from 35.57% (Bowtie2) to 41.81% (BWA-MEM). However, the mapped sequence depth variation was minimal for all aligners (Table 2). In addition, an extended series of 441 independent read alignments was performed for all 21 inbred lines, using three different parameter settings and was evaluated to reveal the most reliable setup (Fig 1A, see Materials and Methods). The agreement between the different read alignment methods was analyzed by calculating those reads that were mapped by the majority of tools, as well as the number of reads mapped by at least one other alignment method (S1 Fig). For all seven evaluated tools the constructed read alignments reached high quality.

**Table 2:** Evaluation of seven read alignment methods. In this broad evaluation 21 inbred maize lines were included. Seven independent read alignment methods were utilized in three different parameter settings. For each alignment methods the best parameter setting is shown with respect to the highest number of reads mapped on target.

Read alignment method	# mapped reads *	Mapped reads [%] *	# reads mapped on target (captured regions)	Reads mapped on target [%]	25 pctil of reads mapped on target [%]	75 pctil of reads mapped on target [%]	Reads mapped by majority (>4) of tools [%]	Reference
**Bowtie2**	9,957,961	95.66	4,182,513	40.18	35.57	45.89	95.48	[21]
**BWA MEM**	10,359,385	99.52	4,352,690	41.81	36.47	47.84	98.83	[22]
**BWA SW**	10,275,404	98.71	4,314,371	41.45	35.77	47.79	98.16	[23]
**CLC mapper**	10,309,608	99.04	4,299,431	41.30	36.35	47.16	98.55	[24]
**NextGenMap**	10,285,973	98.81	4,306,285	41.37	36.49	47.33	98.38	[25]
**Smalt**	10,374,995	99.67	4,322,142	41.52	36.50	47.43	98.83	[26]
**Stampy**	10,389,771	99.81	4,328,226	41.58	36.61	47.45	98.87	[27]

*Number of total reads is 10,409,726

However, our results revealed a sub optimal performance of the tools NGM and Smalt using standard parameter settings. Furthermore, our results indicate that Bowtie2, being one of the most widely used read alignment tools for Illumina sequences, performed less optimal utilizing 454 sequence reads. Thus, we emphasize that read alignment tools and parameter settings should be carefully assessed. Finally, we conclude that BWA-MEM performed best across all genotypes resulting in high confidence of sequence alignments (99.99% of aligned reads were in agreement with another alignment method) and with premier exactness in terms of total aligned reads (99.74%) and reads aligned on target (41.81%).

To evaluate the impact of read alignment on the quality of variant calling, the results of all seven read alignment tools (BAM files) were processed by the all variant calling methods on a randomly selected inbred line (NC358). For this evaluation, we analyzed 504 possible combinations of read alignment and variant calling tools (each individual method was applied in the three parameter settings). Ten incompatible combinations were spotted, which lead to failures (no results obtained) and the overview of all true positive sites detected in each of the individual approaches is depicted in Fig 1B. To determine the impact of read mapping, we compared the merits of each variant caller using different read alignment results as inputs. Using the 504 combinations of read alignment and variant calling methods, we analyzed how the number of successfully detected true positive sites varies with application of different read alignment methods. The observed variability (detected range of true positive sites for a particular variant caller) was, on average, 16.41% (maximum 26.15%) per variant calling method (S2 Fig). The analysis was most consistent for CLC, with only about 5% variability, whereas SNVer was the most variable across different read alignment tools. We then checked the quality of the allele calls obtained using the various tool combinations by comparing them to the genotyping data of the maize 50k SNP array (S2 Table), again using inbred line NC358 as an example. An average genotype concordance of 97.76% was observed, ranging between 95.77% and 98.92% for the various tool combinations. In addition to being the best performer with respect to the number of aligned reads, BWA-MEM also performed best in this analysis. Consequently, BWA-MEM was selected as the preferred tool for subsequent mapping of our 454 maize sequence data.

### *De novo* assembly of captured sequence reads

Quality trimmed 454 reads, totaling ~3 Gbp of sequence, were used to perform *de novo* assemblies, individually for each inbred line applying Newbler (version 2.6, 454 Life Sciences, Basel, Switzerland) with the default settings. Overall, the number of contigs per inbred line ranged from 8,512 to 19,515, and the contig sizes across all inbred lines ranged from 300 bp to 16,184 bp (S3 Table). A total of 15,461 assembled contigs were obtained for the B73 sequences, with a median size of 903 bp and the largest contig encompassing 8,827 bp; this resulted in 14 Mbp of non-redundant sequence. *De novo* assembly of reads from the other genotypes revealed that some of the targeted gene sequences were not completely covered, resulting in more fragmented assemblies.

### Evaluation of variant calling methods

In previous studies, a low concordance of variant calling algorithms was observed when comparing independent variant calling methods [19,20,28]. This can be due to a number of reasons, such as the use of different internal cut-off values, filtering, or variable incorporation of parameters. Further development of variant calling methods is mainly driven by the motivation to implement novel approaches that is faster and more sensitive than concurrent tools. In general, variant calling refers to a Bayesian-based algorithm in order to predict the consensus genotype. Although these are widely used standard methods, different tools incorporate different information to determine the corresponding genotype. Several important metrics exist that describe the quality of a detected SNP; these include ‘strand bias’, the phred-scaled ‘quality score’, the neighboring quality score (NQS), and the coverage filter. The usage of post-filtering is often recommended over the use of internal hard cut-off values by many variant calling tools [29,30]. We therefore decided to employ variant calling at low stringency utilizing the default settings.

To estimate the false positive rates of various SNP discovery protocols, a defined set of verified polymorphic positions was compiled by extracting SNPs from several control data sets (50k, GBS, RNAseq, and HapMap2), which are located within our target regions. These were used to assess the sensitivity (S_e_) and specificity (S_p_) of eight variant calling methods (SAMtools [31], VarScan2 [32], CRISP [33], CLC find_variations [24], FaSD [34], SNVer [35], VCMM [36], and Freebayes [30]). This evaluation involved two approaches, the first of which involved a single inbred line (NC358) for which an in-depth evaluation was performed using various custom, as well as standard, parameter settings for read alignment and variant calling methods (see Material and Methods). The second approach included all 21 inbred lines, applying the standard settings. For the in-depth evaluation, we used the raw variant calling (no filtering) of candidate sites to assess the performance of the detection process without any bias of posterior filtering. Using this method, we observed that the largest number of called variant positions (VP) was observed for FaSD and SAMtools, whereas CRISP, VarScan2, and VCMM detected lower numbers (Table 3). Further assessment revealed that FaSD and SAMtools display the highest sensitivity in calling true positive sites among all tested control data sets (Fig 1C). However, their settings that allow the calling of a large number of putative VPs come at the cost of precision. This is demonstrated in the F_1_-score, which is defined as harmonic mean between sensitivity and precision. For FaSD and SAMtools, we observed average F_1_-scores across all control data sets of 0.25 and 0.24, respectively. In contrast CRISP (0.59) and VarScan2 (0.58) had the highest F_1_-scores, but at the cost of higher numbers of false negative values.

**Table 3:** Variant detection performance. Comprehensive overview of eight evaluated variant detection tools. Predicted VPs of each variant caller are compared to the four control data sets (50k, GBS, RNAseq, and HapMap2) in terms of sensitivity (‘S_e_’), specificity (‘S_p_’), and the F_1_-score (‘F_1_’). In addition the final set of variants detected in this study (CTD) is showing the proportion each variant caller is capturing.

Variant calling method	#total variant calls	CTD*			50k			GBS			RNAseq			HapMap2		
		S_e_	S_p_	F_1_	S_e_	S_p_	F_1_	S_e_	S_p_	F_1_	S_e_	S_p_	F_1_	S_e_	S_p_	F_1_
	**(NC358)**															
	total		128,064			2,524			9,608			27,620			300,664	
**clc**	639,075	0.766	0.972	0.256	0.714	0.972	0.267	0.629	0.972	0.270	0.721	0.972	0.277	0.426	0.975	0.342
**CRISP**	79,194	0.385	0.998	0.475	0.478	0.998	0.760	0.294	0.998	0.732	0.416	0.998	0.694	0.138	0.999	0.293
**FaSD**	986,133	0.823	0.955	0.189	0.976	0.955	0.194	0.857	0.955	0.199	0.868	0.955	0.207	0.880	0.964	0.457
**freebayes**	309,230	0.717	0.988	0.420	0.695	0.988	0.458	0.504	0.989	0.458	0.616	0.989	0.461	0.326	0.991	0.425
**SAMtools**	1,090,172	0.890	0.950	0.187	0.952	0.950	0.190	0.854	0.950	0.195	0.865	0.950	0.201	0.847	0.959	0.420
**SNVer**	262,246	0.613	0.990	0.403	0.516	0.990	0.460	0.402	0.990	0.458	0.507	0.990	0.455	0.228	0.991	0.346
**VarScan2**	115,455	0.499	0.997	0.524	0.412	0.997	0.707	0.280	0.997	0.688	0.392	0.997	0.659	0.151	0.998	0.327
**VCMM**	121,012	0.413	0.996	0.424	0.321	0.996	0.602	0.204	0.996	0.583	0.310	0.996	0.552	0.109	0.997	0.254
	**(read depth >5)**															
	total		383,145			6,127			26,488			73,891			517,877	
**clc**	716,777	0.777	0.979	0.541	0.772	0.979	0.587	0.653	0.979	0.588	0.732	0.980	0.594	0.559	0.984	0.599
**CRISP**	308,032	0.584	0.996	0.648	0.725	0.996	0.841	0.549	0.996	0.832	0.639	0.996	0.823	0.380	0.999	0.618
**FaSD**	467,723	0.755	0.991	0.679	0.832	0.991	0.764	0.680	0.991	0.765	0.713	0.992	0.763	0.592	0.997	0.759
**freebayes**	579,101	0.524	0.981	0.417	0.725	0.981	0.516	0.529	0.981	0.518	0.528	0.982	0.517	0.546	0.989	0.617
**SAMtools**	904,019	0.939	0.973	0.560	0.985	0.973	0.573	0.946	0.974	0.584	0.927	0.974	0.599	0.910	0.987	0.809
**SNVer**	201,330	0.524	1.000	0.686	0.567	1.000	0.991	0.396	1.000	0.959	0.477	1.000	0.910	0.288	1.000	0.521
**VarScan2**	355,679	0.619	0.994	0.642	0.682	0.994	0.799	0.500	0.994	0.789	0.540	0.994	0.771	0.418	0.998	0.640
**VCMM**	367,675	0.879	0.998	0.897	0.615	0.998	0.952	0.451	0.998	0.936	0.532	0.998	0.911	0.340	0.998	0.645

*'CornFed Target Diversity' (CTD) is the final set of VPs in chromosomes.

With the second, broader evaluation approach, the overall tendencies in terms of S_e_ values were confirmed for the majority of evaluated variant calling methods (Table 3, second part). The highest average sensitivities among all the external control datasets was observed in SAMtools (0.94), FaSD (0.71), and CLC (0.70). This larger dataset (all genotypes) lead to a general increase of the total number of detected variant positions. However, a stringent posterior filtering was applied in order to control for the high number of detected VPs that has already been observed (see Materials and Methods). Consequently, the number of false positive sites decreased, resulting in an increase of the F_1_-score, and a subsequent improvement of the overall reliability of the prediction.

This extensive investigation of multiple variant calling methods revealed that that none of the evaluated methods completely captured the complete set of VPs. The low concordance between variant calling methods further highlights the benefit of applying multiple independent tools to obtain a conclusive diversity set. As others have noted [37], VPs detected by multiple tools have a higher validation rate than caller-specific VPs. In that respect, our analysis provides supporting evidence and thus, the inclusion of multiple variant calling methods to improve the validity in the final called SNP set is recommended. We balanced our cutoff to call a specific variant site confident when three independent variant calling methods support the prediction. The cutoff value used in our study was achieved by an analysis of F_1_-scores from different settings that revealed a peak of the F_1_-score at value of three. With this approach we gained confidence and establish a positive validation, without discarding too many SNP candidates by an over-rigorous setting (S3 Fig). Thus, we demonstrate the advantageous effect of applying multiple variant calling methods.

### Large-scale sequence polymorphism discovery

**Fig 2:**
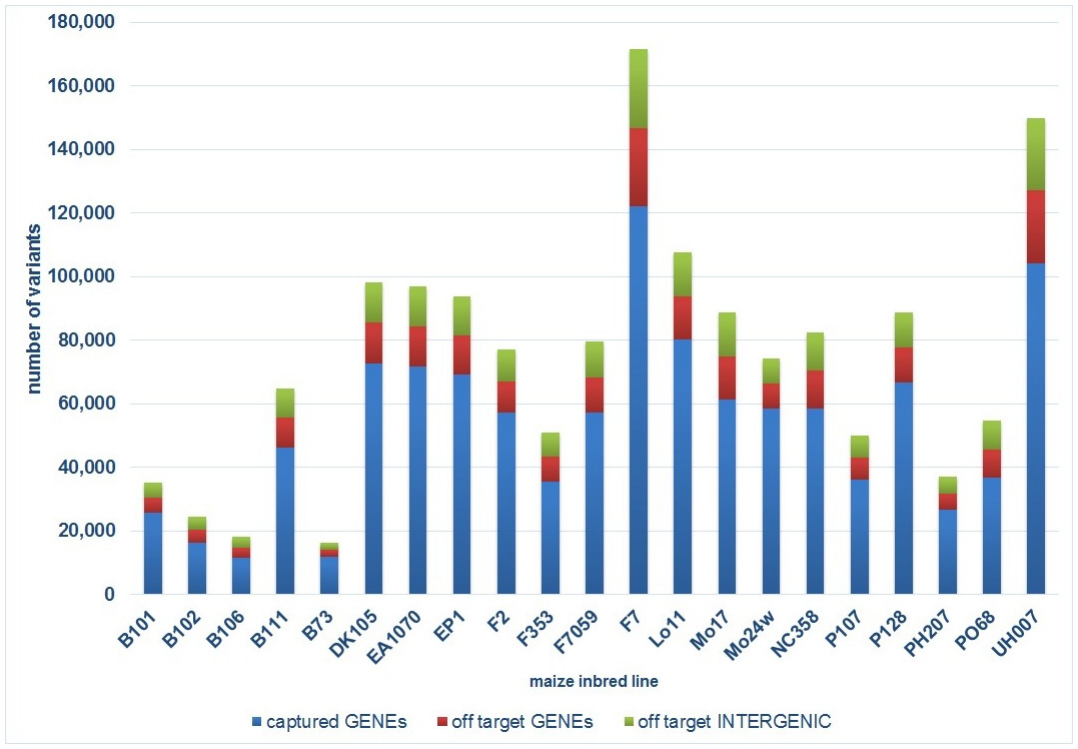
Global diversity classification per genotype. Variant positions in captured genes (on target) identified after basic filtering (at least 5-fold coverage of read depth at SNP position) and off-target regions for different maize inbred lines.

A collection of approximately 4.8 million VPs was called across all 20 non-B73 inbred lines when these were compared against the B73 reference sequence in the initial phase of the discovery process. The inclusion of a pre-filtering step, requiring minimal coverage of at least five reads per VP, led to a global total of 696,665 variants, including ~10% insertions and deletions (INDELs). However, a final list of approximately 42,000 INDELs was obtained after discarding positions overlapping with homopolymers (>7 bp) in the maize reference sequence, which are error prone in 454 sequences [38]. We found that 71% of detected VPs were located on target, while 29% were off-target. Off-target positions were uniformly distributed among all ten maize chromosomes, and several variants were detected in mitochondrial (249) or plastid (536) sequences. Of all SNPs and INDELs called as off-target, 74.9% and 68.2%, respectively, were located in genic regions (off-target captured genes), likely mapping to the paralogous sequences that are closely related to the target genes (Fig 2).

To further increase the reliability of *in silico* VPs prediction, the results of multiple prediction tools were combined for validation. This combinatorial approach required that a VP be predicted by at least three independently performed variant calling methods (S3 Fig). Since different variant calling tools use different quality score scales to measure the significance of a prediction, we normalized the score and applied a generalized minimal threshold of 0.4, where 1.0 is the maximal score of the particular tool. A total of 383,145 variants (including ~91% SNPs and ~9% INDELs) were called across the 20 non-B73 inbreds, with an average of 45,594 SNPs per line (Table 4). In the following, we refer to this variant set as ‘CornFed Target Diversity’ (CTD), emphasizing that the observed diversity is confined to the investigated set of target genes and closely related sequences across the studied lines. Out of these, only 127 SNPs were called in all non-B73 inbred lines (Table 4). A total of 18,489 insertions and 23,039 deletions were called across theses inbred lines, with global average of 1,925 and 2,469, respectively, per inbred line (Table 4).

**Table 4:** SNP functional class membership. Re-sequencing and variant calling within 21 maize inbred lines discovered 383,145 high quality candidate SNPs (complete S12 Table). Table displays the five least diverse (green) and five most diverse (blue) inbred lines, also differentiating between the dent [‘D’] and flint [‘F’] inbred lines. The intersection (‘**∩**’) was analyzed in four settings (5, 10, 15, and complete), indicating that the variant site is present in at least the respective number of genotypes.

Maize inbred line	B106 [D]	B102 [D]	B101 [D]	PH207 [D]	P107 [D]	DK105 [F]	EA1070 [F]	Lo11 [F]	F7 [F]	UH007 [F]	∩ 5+	∩ 10+	∩ 15+	∩_complete_	∪_complete_
**Total variants**	11,386	16,190	22,215	22,624	31,602	61,253	63,533	67,983	74,820	96,158	54,212	11,721	2,209	127	383,145
**Homozygous**	6,308	9,467	13,104	13,907	20,299	40,688	42,465	46,490	49,292	56,986	10,124	1,620	231	1	265,728
**Insertions**	385	487	849	953	1,234	2,830	2,773	3,392	3,317	3,728	1,958	238	27	0	18,489
**Deletions**	437	621	1,128	1,062	1,599	3,465	3,496	4,000	4,025	5,547	2,603	402	68	8	23,039
**Variants overlapping target region**	6,723	10,412	15,847	16,092	22,143	44,418	44,463	47,689	49,992	61,059	10,879	2,201	451	8	259,547
**Variants overlapping 50k** [5]	174	272	355	367	550	976	907	1,092	1,049	1,121	305	77	16	0	3,822
**Variants overlapping GBS** [39]	442	582	864	1,043	1,196	2,520	2,373	3,063	2,948	3,120	640	151	27	0	12,432
**Variants overlapping RNAseq** [40]	1,544	2,624	3,580	3,639	5,155	9,384	9,186	10,508	10,390	11,544	2,566	690	140	1	41,226
**Variants overlapping HapMap2** [41]	4,726	7,472	10,452	10,651	16,260	32,009	33,838	35,868	38,136	49,119	8,647	1,594	239	1	190,347
**Functional classes**															
**SNPs in exons**	4,755	6,672	9,776	10,204	12,268	24,432	23,091	26,167	27,811	31,372	15,586	3,776	664	13	86,599
**Silent mutation**	7,635	11,001	14,982	14,984	22,711	44,794	48,033	50,558	55,745	75,227	38,050	7,615	1,428	105	299,126
** SNPs in introns**	1,577	2,426	3,360	3,329	5,639	11,259	11,673	11,878	12,866	18,427	2,844	461	78	7	70,218
** SNPs in UTR**	1,844	2,714	3,987	4,638	6,175	13,268	13,967	15,214	15,622	19,050	3,136	523	93	2	89,583
**Non-Synonymous SNPs**															
** non conservative missense**	320	448	591	693	690	1,383	1,295	1,416	1,603	1,836	1,229	286	54	0	8,228
** conservative missense**	1,433	1,863	2,576	2,836	3,103	5,881	5,437	5,968	6,807	7,653	5,388	1,314	217	5	34,457
**Synonymous SNP**	1,845	2,654	3,699	3,708	4,716	8,263	7,936	9,032	9,561	10,202	8,687	2,115	377	7	41,334
**Frame shift**	58	95	142	186	153	404	351	438	489	564	282	61	16	1	2,580
**SNPs located in splice site**															
** variant in splice site** ^**a**^	116	180	276	262	404	701	675	774	820	998	151	35	6	1	4,131
** variant in essential splice site** ^**b**^	27	28	56	48	65	132	143	128	165	211	26	6	1	0	931
** variant in splice donor site**	14	15	22	26	29	44	46	56	63	93	45	10	2	0	364
** variant in splice acceptor site**	8	11	32	26	31	72	59	71	80	92	55	12	4	0	388
**SNP leading to premature terminal codon**	38	48	77	65	64	159	141	161	168	216	110	13	5	0	1,170
**SNP eliminating terminal codon**	3	8	14	7	21	33	26	35	44	37	29	10	6	0	155
**Transition** ^**c**^	6,957	9,731	12,713	13,214	17,792	33,410	34,826	35,757	40,809	54,148	8,120	1,631	367	13	229,728
**Transversion** ^**d**^	2,985	4,474	6,040	5,920	8,800	17,138	17,877	19,445	20,992	26,465	4,400	830	171	7	106,399

^a^ SNP is located 1–3 bases into an exon or 3–8 bases into an intron

^b^ SNP is located in the first two or the last two bases of an intron

^c^ transistion (A <-> G, C <->T)

^d^ transversion (C <-> G, A <-> C, G <-> T, A <-> T)

^∩^ intersection using the complete SNP data set of 21 genotypes

^∪^ union using the complete SNP data set of 21 genotypes

Finally, to estimate the variant caller performance, we determined the fraction of the final list of variants (383,145 sites) detected by each variant caller in our data set. This was achieved by looking at the sensitivity (S_e)_ of each variant calling method in the CTD set (Table 3). According to these criteria, SAMtools (0.94) was ranked first, followed by VCMM (0.88), CLC (0.78), FaSD (0.76), VarScan2 (0.62), CRISP (0.58), Freebayes (0.52), and SNVer (0.52), in the detection of VPs that were ultimately confirmed in our CTD set (Table 3). The most balanced performance was observed for VCMM, which simultaneously displayed a relatively small number of total VPs and an exceptionally high number of true positive sites. Consequently, VCMM (0.90) was ranked first with respect to the F_1_-score, followed by SNVer (0.69), FaSD (0.68), CRISP (0.65), VarScan2 (0.64), SAMtools (0.56), CLC (0.54), and Freebayes (0.42).

Using our combinatorial variant calling approach, we achieved a new level of confidence for SNP calling in inbred maize lines. The comparison of this method to the stand-alone application of each single variant caller showed that purely filtering by read depth would result in almost double the number of VPs (681,993). The majority of additional VP candidates (299,971) was observed by only a single tool and in only a single genotype (56.0%). In addition, 24.1% were solely predicted by a single tool, and another 11.2% were observed only in a single genotype. Although these sites might be true rare allelic variants, the probability of erroneous calls is much higher due to very low statistical evidence [42]. The analysis of polymorphic information content (PIC), as defined previously [43], revealed a very low PIC value for removed positions. With a median value of 0.07, only 6,982 sites (2.33%) were characterized with sufficient values (0.2–0.5). Consequently, these indicate a lower reliability, supporting the decision to discard these positions. An alternative option to the applied combinatorial variant calling is to perform a more stringent filtering at higher read depth. Increasing the applied threshold of read depth resulted in a very substantial reduction of detected sites with a high likelihood of losing meaningful VPs. When a read depth threshold of 10 per site was applied, only 31.6% of the final CTD calls were detected. We conclude that the application of a combinatorial variant calling approach offers a higher reliability than stand-alone methods and thus is a useful option for diversity calling, especially in low-coverage sequencing.

### Genetic relationships among the 21 inbred maize lines

**Fig 3:**
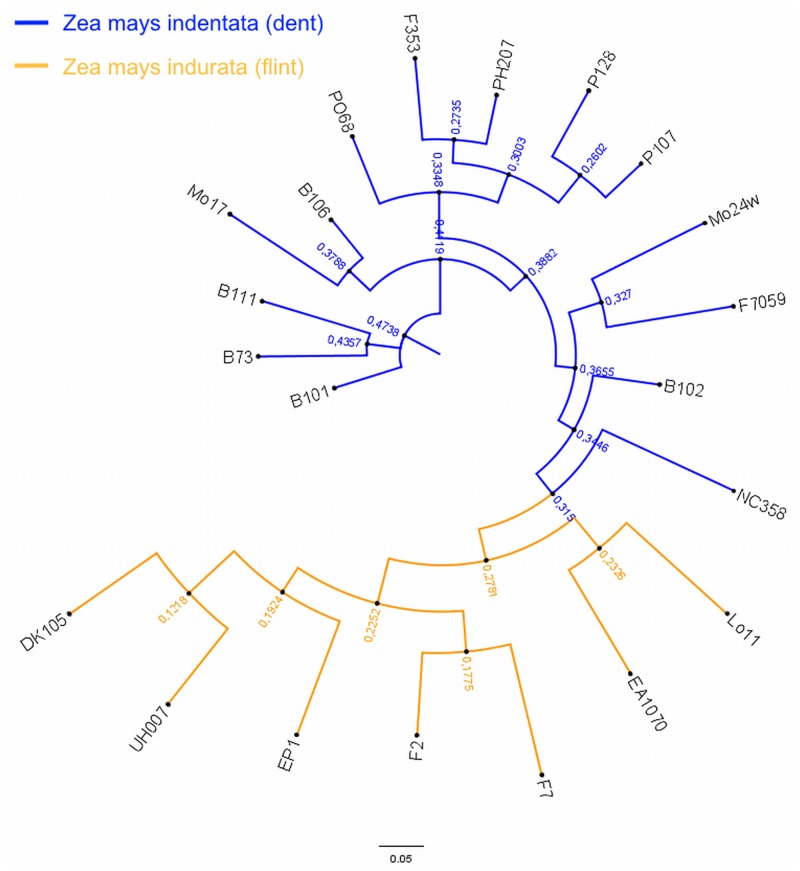
SNP based phylogenetic tree. Phylogenetic tree depicting the SNP distance between 21 maize inbred lines, emphasizing the diversity in this collection. Over 265,000 homozygous SNP calls have been processed to construct this dendrogram.

The genetic relationships among the 21 inbred maize lines were assessed based on the genetic variation across the re-sequenced genes and the corresponding SNP profiles. SNP profiles clearly differentiated the inbred lines into the two major European gene pools (Dent and Flint, Fig 3). The flints were further differentiated into two groups, with lines originating from Spain in one group (except EP1), and lines originating from France or Germany in the second, larger group. This larger group was further differentiated into three subgroups, representing lines originating from Germany, Spain, and France. The dent lines were further differentiated into three major groups, the first consisting of USA lines, the second containing lines from Germany and France (except PH207, which is a USA material), and a third group consisting of lines from Canada, USA, and France.

**Fig 4:**
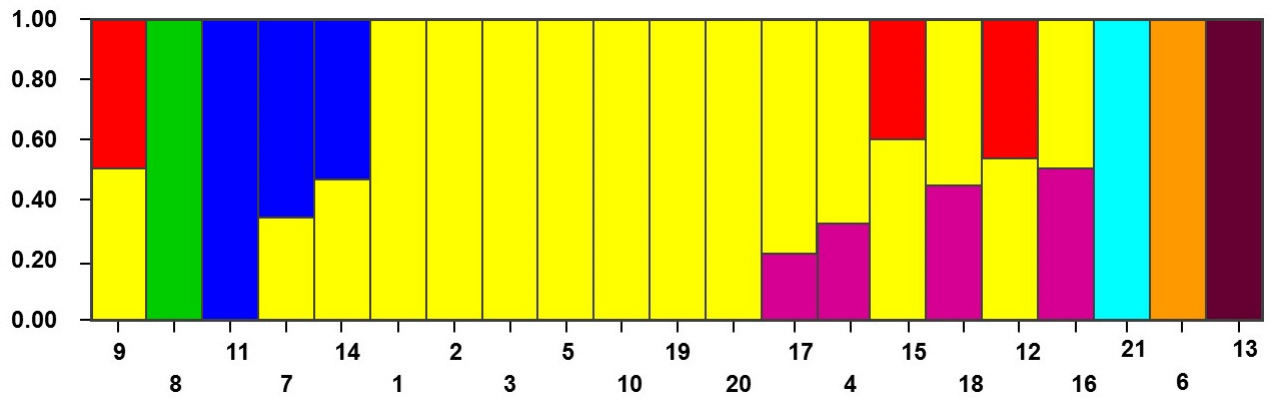
Bar plots of the STRUCTURE analysis. Each of the 21 maize inbred lines is represented by a vertical bar, partitioned into K = 8 colored segments that designate the fraction of each population estimated to belong to the inferred subgroups (Population ID corresponds to S1 Table).

STRUCTURE analysis predicted K = 8 as the optimum number of sub-populations, revealing that at least eight distinct groups exist in the studied inbred lines (Fig 4 and S4 Fig). Some groups displayed heterogeneity, comprising a sizable portion of another group; however, most inbred lines originating from the USA clustered together in one group (yellow-filled bar). In regards to the two main gene pools (Dent and Flint), the majority of flints, except those originating from France, were classified into four distinct groups, each consisting of an individual inbred line. The flints originating from France contained a significant portion of USA dent materials. On the other hand, dent materials (except F7059, originating from France) display heterogeneity, comprising a portion from at least one of the four dent groups.

### Functional analysis of SNPs

To exclude most erroneously called VPs from the functional analysis, only the final set of variants comprising 383,145 polymorphic positions (including INDELs), which was validated *in silico* by the combinatorial variant calling approach, was considered. A total of 229,728 and 106,399 SNPs were predicted to be transitions or transversions, respectively (Table 4). Of these, 86,599 (22.6%) were located in exons, with a global average of 11,406 SNPs per inbred line (Table 4). The majority of SNPs (78.1%) represented silent mutations. However, a substantial number (42,685 SNPs) were non-synonymous (nsSNPS), with 8,228 SNPs classified as non-conservative missense and 34,457 SNPs classified as conservative missense. A total of 2,580 INDELs were annotated as frameshift mutations. In addition, 4,131 VPs were detected in splice sites, including 931, 364, and 388 VPs that were classified as variants in the essential splice site, splice donor, and splice acceptor, respectively. We further found 155 SNPs that were predicted to eliminate a terminal stop codon and 1,170 SNPs that inserted a premature stop codon.

**Fig 5:**
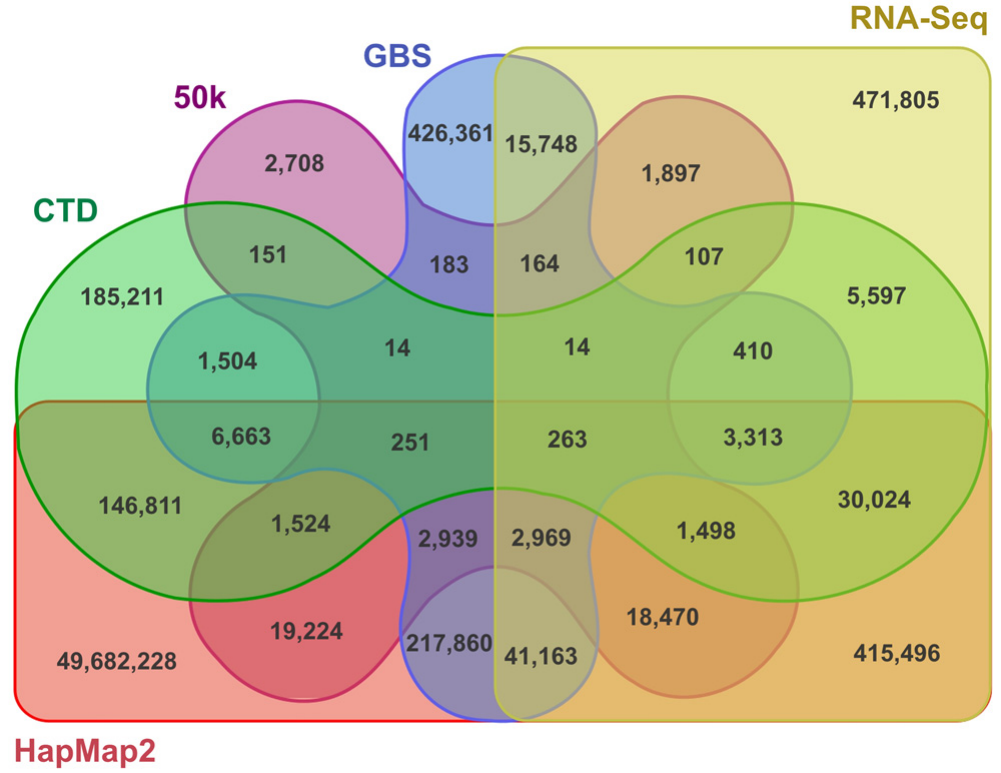
Venn diagram of marker/variants in maize. Illustration of the overlapping intersections between four diversity data sets (50K, GBS, RNAseq, and HapMap2) and the ‘CornFed Target Diversity’ (CTD).

To determine the number of novel variants detected in this study, we calculated the overlap between variants detected in this analysis and those listed in previously published maize variant resources. Using this comparative analysis, 3,822 (7.3%) VPs from the 50k array, 12,432 (1.7%) from the GBS, 41,226 (4.4%) from RNAseq, and 190,347 (0.3%) positions from the HapMap2 data set were ascertained to be consistent with those identified here. Thus, the majority (~52%) of *in silico* predicted variants were found to corroborate those available in public data sets. However, a large proportion of variants were newly detected in this study (Fig 5), leading to a set of 185,211 variant positions that have not yet been documented.

### Gene diversity and functional annotation of target genes accumulating radical mutations

**Fig 6:**
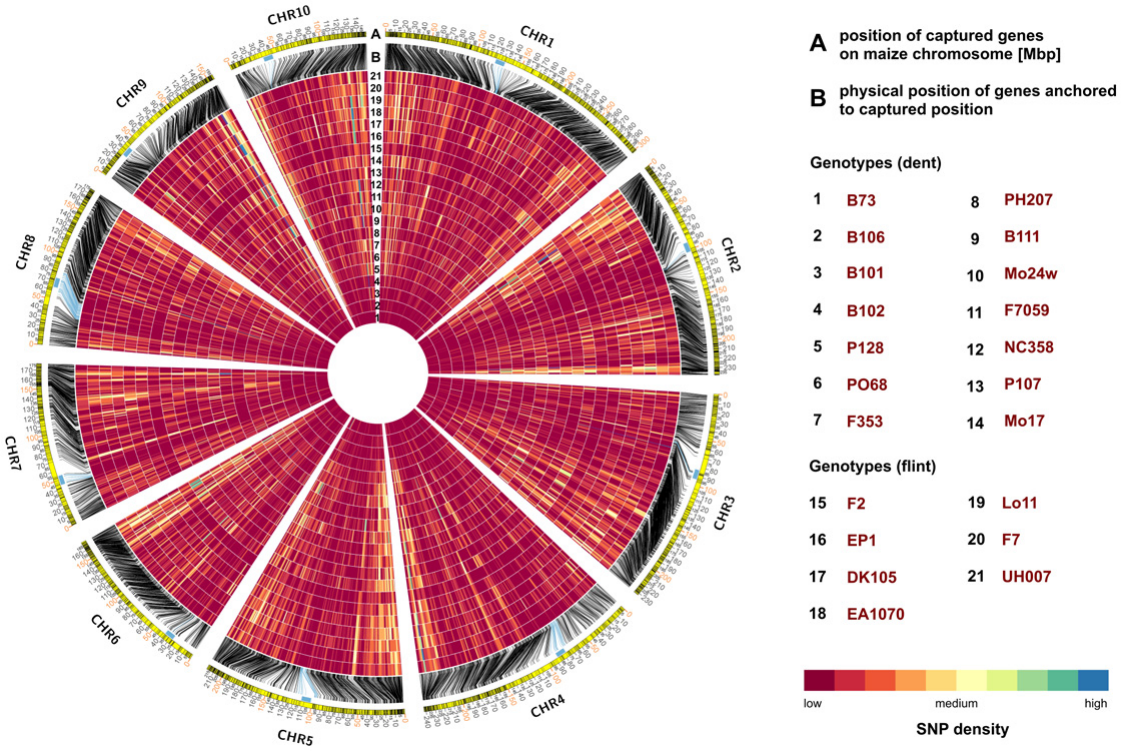
Captured target gene diversity in 21 maize inbred lines for biomass production and composition. **(A)** The outer circle illustrates, with dark connecting lines, the location of the 4,372 target genes in the 10 maize chromosomes. The inner circles (heat maps 1–21) represent the 21 (ordered in dent and flint inbred lines and subsequently with increasing diversity) inbred lines and their corresponding SNP density, scaled according to the physical position of the target genes in the B73 genome reference **(B)**. In total, 1 million of the detected high quality SNPs are integrated. Putative regions of high diversity are represented in blue, and regions with low accumulations of mutations are colored red. Centromere positions are indicated with light blue bars adjacent to the outer circle.

To measure gene diversity across the 21 inbred lines using the final set of variants (CTD), a heat map illustrating the diversity of captured genes in these lines in comparison to the B73 reference line was constructed (Fig 6). We observed that the majority of genes displayed low diversity in terms of nucleotide changes. However, interesting patterns of divergence between the elite flint and dent inbred lines were observed near the ends of chromosomes in regions of high diversity. Specifically, flint inbred lines displayed high nucleotide variation on chromosome 3 at physical position 109 Mbp, while dents had only minor changes here. Conversely, an opposite pattern was observed on chromosome 8 at physical position 114 Mbp, where dent inbreds were found to be more diverse, as compared to B73, than flint inbred lines (S6 Fig).

As stated above, a total of 86,599 unique SNPs was detected in exons (coding SNPs), with an average of 11,762 SNPs per inbred line (S4 Table). The majority (82.0%) of these coding SNPs were classified as rare variants, as they occur in genes affecting five or fewer inbred lines. Only 2,843 coding SNPs were detected in more than half of the inbred lines, 13 of which were found in all 20 lines. The number of genes per line with at least one coding SNP ranged from 1,025 to 3,446 out of the 4,484 re-sequenced genes, with an average of 2,293 genes per inbred line (S5 Table). In absolute numbers, most coding SNPs were synonymous. In total, 41,334 positions that are polymorphic in at least one inbred line are classified as synonymous, whereas 8,228 are associated with more drastic effects and are annotated as ‘non-conservative missense’. The number of nsSNPs in each line ranged from 225 to 2,089, with an average of 1,115 per inbred line. The number of genes with at least one nsSNP ranged from 182 to 1,113, with an average of 625 genes per inbred line. A total of 1,488 genes had a unique nonsense SNP detected in at least one inbred line. In each line, the total number of genes with at least one nonsense SNP ranged from 211 to 590, with an average of 317 genes per inbred line, while the number of nonsense SNPs ranged from 244 to 1,638, with an average of 886 per inbred line. The overall distribution of nonsense SNPs suggests that they are rare variants, with the majority (82.6%) occurring in five or fewer inbred lines.

In total, 45.2% of captured genes were found to harbor at least one nsSNP (S6 Table). Of these, a set of 67 genes was identified as potential candidates for manipulation of biomass production. These were selected by checking the two highest biomass yielders (F2 and F7) and the two lowest biomass yielders (B111 and EA1070; S7 Table) and selecting genes that carry nsSNPs in only one of these two groups (S6 and S8 Tables), either only in the low or only in the high biomass yielders. We observed that 46/67 candidate genes carry nsSNPs only in the high yielding lines, suggesting the modified alleles may be associated with increased biomass. The other 21 genes contained nsSNPs in low yielding lines, perhaps revealing the opposite association. For example, GRMZM2G024374, a 6-phosphofructokinase involved in the glycolytic pathway, contained nsSNPs in the low yielding inbred lines, but none in the high yielding ones. Two genes, AC217050.4, encoding a terpene synthase, and GRMZM2G034069, which is involved in brassinosteroid biosynthesis, contained radical mutations in high yielding lines, but not in low yielding lines.

### Presence/absence variation (PAV)

On average, 93 genes (2.2%) per line were classified as completely missing (no read aligned either partially or fully to the target gene sequence), and this ranged from 56 to 169 genes across all studied lines (S9 Table). We further observed that deletion of a given gene relative to the B73 reference was not distributed as simple presence/absence across the studied inbred lines nor was this the case for different genes within an inbred line. If a maximum coverage of 10% of a target gene relative to its sequence in B73 was allowed (90% or more not covered), on average 118 genes per line were classified as missing, ranging from 83 to 215 genes across all studied lines. By relaxing the threshold to a maximum of 20% coverage of a target gene sequence (80% or more not covered with reads), 177 genes were classified as missing, ranging from 114 to 313 across the studied lines. We ultimately declared a gene to be present in a given inbred line if more than 25% of its sequence length (according to the re-sequenced B73) was covered by reads. With this threshold, an average of 220 genes were classified as missing, ranging from 138 to 420 across all studied lines.

## Discussion

Maize is a critical crop for human food and livestock feeds; it contains a large and complex genome and exhibits high levels of both phenotypic, and genetic, variation. In this study, we designed a sequence capture assay to identify variable loci within inbred maize lines that may be involved in growth and biomass accumulation. We observed that a significant amount of our 4,648 studied genes accumulated VPs with a substantial proportion of non-synonymous mutations that might affect the functional integrity of these genes. In addition we observed with a comparative sequence analysis further differences between the studied inbred lines regarding presence and absence of genes that may provide further candidate loci for extended analysis. We are confident that with our in-depth evaluation of computational methods we established a useful approach for diversity studies.

### Use of sequence analysis tools

It has previously been observed that variant calling concordance can vary markedly between different tools [17,36,44]. In agreement with these findings, we also observed a substantial incidence of discordant SNP calls. Therefore, we argue that a combinatorial variant calling approach that uses different SNP prediction tools is a prospective practice to achieve results with high confidence. Our evaluation of mapping tools further demonstrated that use of non-optimal read alignment tools might result in a loss of up to 26% of VPs inherent in the analyzed inbred lines. Thus, read alignment has a strong impact on variant calling, and the use of an optimal procedure is of central importance. A great potential exists for re-alignment methods that optimize a constructed alignment with the aim of achieving improved and more reliable SNP calling. Further, recalibration of base quality and optimization of read positioning can also have a large impact on prediction correctness [45,46].

### Evaluation of variant calling methods

In this study, we evaluated the merits of various methods for calling high quality variants in 454 sequence data. Based on the final list of variants (383,145 sites; Table 3), the different variant callers varied in performance and displayed comparable patterns of performance values across the four control data sets (50k, GBS, RNA-Seq and HapMap2), in both the overall dataset analysis using standard settings and the results of an in-depth analysis performed on genotype NC358 using variable parameter settings. In regards to the three performance measurements (S_e_, S_p_, and F_1_), the different tools were differentially ranked and displayed comparable ranking patterns when checked with the four control data sets (Table 3). On average, SAMtools (0.94) showed the best sensitivity. Overall, we observed that the average values of these measurements provide a good approximation of global performance. We note that while tools with high numbers of detected VPs have a higher probability of including known true positive VPs, these also predict more false positive sites. However, this does not necessarily reflect the overall exactness of the prediction. To measure this, we compared the specificity (S_p_) of the different tools and found that, in this case, the variant callers with low numbers of detected VPs performed best, with SNVer having the best specificity. The F_1_-score is an indicator of the optimal combination of sensitivity and precision. Regarding the external control data sets 50k, GBS and RNA-Seq, that have relatively small overlaps with the sequence variants detected in this study, our studied diversity panel assessment revealed that the variant calling methods with lower number of predicted VPs (CRISP, SNVer, VarScan2, and VCMM) showed better performance regarding the F_1_-score. Conversely, FaSD, Freebayes, and SAMtools showed better performance for the large HapMap2 control dataset. The sensitivity analysis revealed lower values than one would expect for high correctness. However, it is important to note, that a predicted VP, which is not present in the control data set, is not necessarily a false positive. The corresponding VP may have been excluded from the genotyping array design, or the applied variant calling method was not able to detect that VP (i.e., intronic SNPs are not represented in RNAseq). According to our evaluation of these methods using the sequence data from 21 inbred maize lines, the most reliable polymorphism prediction is achieved when multiple variant callers are cross validated. The resulting combinatorial variant calling approach performed with higher exactness. With respect to the evaluated eight variant calling methods, we observed best results when a VP was called by at least three methods. Consequently, we are confident that our final diversity set has a higher validity than would be the case if only an individual variant calling method were used.

### Large-scale sequence polymorphism discovery

A comprehensive catalogue of genetic variants, including SNPs, INDELs, CNV, PAV, and common and rare variants is an essential resource for studies aimed at identifying those variants that affect phenotypic expression. In this study, we detected a large number of SNP and INDEL sequence polymorphisms. Although they are present at lower rates than SNPs, small INDELs represent a functionally important type of genomic variation [47,48]. In addition we detected numerous cases of PAV among the studied inbreds, which is an important component of genetic variation and hitherto largely untapped. The intraspecific variation of gene content observed even in the limited data set of this study (addressing 4,648 genes in 21 maize inbred lines) indicates that it represents a highly relevant source of genomic variation that can potentially contribute to population’s ability to adapt to environmental changes. Therefore, it is important to gain further knowledge of presence absence variation to deepen our understanding of genome diversity and to support the identification of functional variation in traits of interest. We also identified 185,211 novel VPs not present in public datasets, which can be of interest for the design of SNP markers to study variable SNPs with potential functionality or functionally variable haplotypes.

### Relatedness of the studied inbred lines and chromosomal distribution of the detected sequence variation

The genetic relationships between 20 inbred maize lines and the B73 reference genome were investigated using the CTD set. A neighbor-joining dendrogram based on SNP profile splits into two main branches, corresponding to the dent and flint groups (Fig 3). These detected groupings were expected and consistent with the historical data on the origin of the inbred lines (S7 Table). For example, UH007 and DK105 are University of Hohenheim breeding materials (flint gene pool). Bouchet et al 2013 working a sample of 375 maize lines representing the worldwide diversity found a similar [49]. The groupings were confirmed by the genetic structuring of the inbred lines, with structure plot giving the position of the inbred lines according to either their known or apparent origin populations (Fig 4 and S7 Table). A majority of the USA lines displayed allelic similarity, with the B series grouping closer to B73 than the others, as would be expected since the name B series (including B73) denotes an apparent origin from Iowa state maize populations (S7 Table). Conversely, the lines originating from France or Germany displayed higher differentiation relative to B73. Overall, the relationships among the inbred lines relative to B73 were reflective of their geographical origin, and thus perhaps a common ancestry, rather than the genetic pool (dent and flint). On the other hand, dents from USA seem to have contributed largely to the genomes of dents in other regions.

Visualisation of SNP density distribution revealed high variation along the ten maize chromosomes. A higher differentiation between the inbred lines (Fig 6) is found at the distal ends, perhaps due to high recombination rates expected in these regions, whereas regions closer to the centromere show lower levels of diversity (S5 Fig). This is in agreement with other observations showing higher conservation at the centromeres [50].

Beyond this overall distribution, several interesting patterns of variation in the density of sequence divergence were observed when the chromosomes were analyzed in bins of 50 kbp size and screened for regions that exceed the 60% quantile of the maximal VP density (across all inbred lines for particular 50kbp) in at least five genotypes when compared to all other genotypes. Discovered regions are indicated by bars in the second outer circle (S6 Fig). A clear differentiation between dent and flint lines was found on chromosome 3 around the sequence position 109 Mbp. Within this genomic region, a high SNP density was observed in the flints, while a significantly lower SNP density was observed in the dents. The opposite pattern was found on chromosome 8 (114 Mbp), where the dent inbred lines show significantly higher diversity than the flint inbred lines. In addition to the detected loci specific for the dent or flint populations, we found 100 loci of high SNP density. These regions that exceeded the 60% quantile in at least 5 inbred lines sum to ~1.5 Mbp of genic sequences.

### SNP annotation

The goal of annotating SNPs is to provide a reference as to which ones may be functionally relevant. Our detected SNPs were assigned to a diverse range of functional classes, with the majority classified as silent mutations. These may have no or little effect on the phenotype. Among all classes of SNPs detected, nsSNPS are the most likely candidates for causal mutations, as they could alter the structure and function of relevant proteins. Such genetic variation may thus account for substantial trait variation in the studied lines. We observed an average of 984 nsSNPs per line in gene coding regions (Table 4), with a total union of 8,228 nsSNPs across all inbred lines. In humans, nsSNPs in gene coding regions could account for nearly 50% of the known genetic variations linked to human inherited diseases [51]. Thus, a larger effort would be warranted to study potential links between the identified nsSNPs and trait variation in maize, and to determine and how they affect the regulation of biological pathways and processes. The 1,170 SNPs that create premature stop codons and the 4,131 SNPs that are predicted to affect splicing can also be predicted to have particularly pronounced effects (Table 4). SNPs selected or prioritized in this way would be highly preferred marker sets to be subjected to association studies using suitable larger populations such as the CornFed Dent and Flint panels for which very substantial genotype and phenotype information has already been collected [52,53].

### Gene diversity and functional annotation of target genes accumulating radical mutations

The majority of genes displayed low sequence polymorphism across all studied lines, with an average of 10 VPs per target gene. This translates to a global average SNP density of 1/639 bp within these target genes, suggesting that the majority may be involved in key plant pathways and hence contain a low number nucleotide changes. This correlation between low gene diversity and its importance for the organism has been emphasized in previous studies [54]. Relating SNP densities in exons and in introns, we observed a ratio close to 1 (Table 4). The nucleotide variability in the non-coding part of a gene is expected to be higher than the variability observed in the coding part, because non-coding polymorphic sites are less likely to cause structural changes of encoded proteins that could lead to functional consequences [55]. However, because of the capture array design, which required exclusion of repetitive sequences, we postulate that this ratio is slightly skewed towards an underestimation of polymorphic sites in introns. Previous studies have demonstrated the insertion of repetitive elements into the non-coding sequence of maize genes [56], and consequently, repetitive probes that originate from intron sequences would have been discarded in our scheme, resulting in their consistently lower representation on the array. In order to test this hypothesis, we analyzed the sequence coverage within exon and intron sequences. In all studied maize inbred lines, the calculated coverage in coding regions exceeds the coverage in non-coding gene sequences (on average by ~15.2%). Beside the exclusion of repeats in the design of the array an excess of coverage in coding sequences can also be explained by a higher degree of sequence conservation as compared to the non-coding sequences. High sequence similarity is a requirement for successful sequence capture that would tolerate only a minute level of nucleotide variation. These results highlight the fact that the properties of the capturing system needs to be considered for correct interpretation of results, especially if different levels of sequence conservation can occur and they indicate a better performance of the approach in the analysis of coding versus non-coding sequences.

A much more detailed perspective can be considered on the gene level when utilizing the complete diversity files (S1 File) or the list of genes with radical mutation (S6 Table). These genome regions, which include 344 of the target genes, can be regarded as candidate loci for further studies aiming at linking genetic variation to phenotypic variation related to yield formation / biomass production in maize. A further refinement in the nomination of candidate genes is possible within the existing data set, when the differential occurrence of radical mutations in high and low biomass producing lines is taken as selection criterion. Although, due to the very restricted number of lines taken into account in the example given in this study (2 low and 2 high yielding lines), no statistical support can be given to the relevance of the selected 67 genes (S8 Table), they could be regarded as prime candidates for targeted association testing. This is supported by the suggested roles of some of the encoded gene products in plant growth:

The GRMZM2G034069 gene is involved in brassinosteroid biosynthesis and showed an enrichment of radical mutations in high yielding lines, as compared to low yielding ones. Brassinosteroids are a major hormone class controlling plant growth and development [57]. In contrast, GRMZM2G110881 displayed an accumulation of radical mutations in low yielding lines and not in high yielding ones. It encodes a UDP-glucose 4-epimerase, which converts UDP-galactose to UDP-glucose and is involved in glycosyltransferase reactions in metabolism. GRMZM2G393762, which encodes a pectinesterase, also showed an accumulation of radical mutations in low yielding lines, as compared to high yielding ones. Pectinesterases are involved in plant cell wall modification and subsequent breakdown. Additionally, many genes involved in plant defense reactions (e.g., AC211164.5 and GRMZM2G702176) have accumulated radical mutations in the low yielding lines but none in the high yielding ones.

### Presence absence variation

Another level of genome diversity / sequence variation, which is increasingly recognized and considered as a further potentially important determinant of trait variation in maize [2–4] is presence / absence variation. Numerous gene sequences of up to several kilobases in length were found in this study to be absent in at least one of the studied inbred lines. These sequences may be common in the maize population, and thus their absence in the genome of a given line might be a rare variant. Using a threshold cut-off of at least 75% gene sequence loss relative to the reference gene sequence in the B73 genome, 2.2% of the targeted genes were found to be absent in at least one of the studied lines.

When the two lowest and two highest biomass yielders were compared, 30 presence / absence genes of interest were identified (S10 Table). Among them are three genes (GRMZM2G048775, GRMZM2G050829, and GRMZM2G129935), which encode peroxidases and are possibly involved in the betanidin degradation pathway. GRMZM2G048775 and GRMZM2G050829 are present in the genomes of low yielding lines but absent from the genome of high yielding lines, while GRMZM2G129935 is only present in high yielding lines. Plant peroxidases are encoded by a large multigene family, and are known to participate in a broad range of physiological processes, such as lignin and suberin formation, cross-linking of cell wall components, and synthesis of phytoalexins [58]. They are also known to participate in the metabolism of reactive oxygen species and reactive nitrogen species, both of which trigger the hypersensitive response, a form of programmed host cell death at the infection site, associated with limited pathogen development [58]. Most genes in this category were found in the genomes of low yielding inbred lines and absent from genomes of high yielding lines. Other identified candidate presence/absence genes include ones involved in glycerol degradation (e.g., GRMZM2G079100), plant sterol biosynthesis (e.g., GRMZM2G014789), glycogen biosynthesis (e.g., GRMZM2G481027, also called glycogenin glucosyltransferase), acyl carrier protein metabolism (e.g., GRMZM2G4181199), and adenosine nucleotides *de novo* biosynthesis (e.g., GRMZM2G470035) pathways. Additionally, an enzyme encoded by GRMZM2G079100 (sn-glycerol 3-phosphate:ubiquinone-8 oxidoreductase) is essential for post-germination growth and seedling establishment [59]; this gene was found to be present in high yielding inbred lines but absent from low yielding lines, possibly indicating the importance of seedling establishment to the final biomass. In addition to the genes showing differential occurrence of radical mutations in high and low biomass producing lines, the differentially present / absent genes share the same level of support and should thus as well be considered with priority in follow-up experiments towards testing their associations with high or low biomass accumulation.

In summary, the targeted re-sequencing of a large list of selected genes across a series of diverse maize inbred lines differing in biomass accumulation revealed a high degree of DNA sequence polymorphism. We were able to narrow down several candidate loci that may play a role in the phenotypic differences between different maize lines, and these results may be considered for future studies aimed at optimizing yield in this important agricultural resource.

## Materials and Methods

### Materials

Maize lines were obtained from the PLANT-KBBE-CornFed project: B101, B102, B106, B111, B73, F353, F7059, Mo17, Mo24W, NC358, P068, P107, P128, and PH207 are from the CornFed Dent panel, and DK105, EA1070, EP1, F2, F7, Lo32, and UH007 are from the CornFed Flint panel [52,53]. These inbred lines were chosen to represent a wide range of biomass production properties. The geographic origin and a short description of the maize inbred lines used in this study are presented in S7 Table.

### Methods

#### Sequence capture array design

The sequence capture array was designed to target the full-length enrichment of gene sequences and was geared for the generation of re-sequencing data for complete gene haplotypes. An inventory of 4,823 candidate genes representing possible targets for modification of biomass accumulation and production, as well as water use efficiency, was compiled from the published literature (S11 Table).

**Fig 7:**
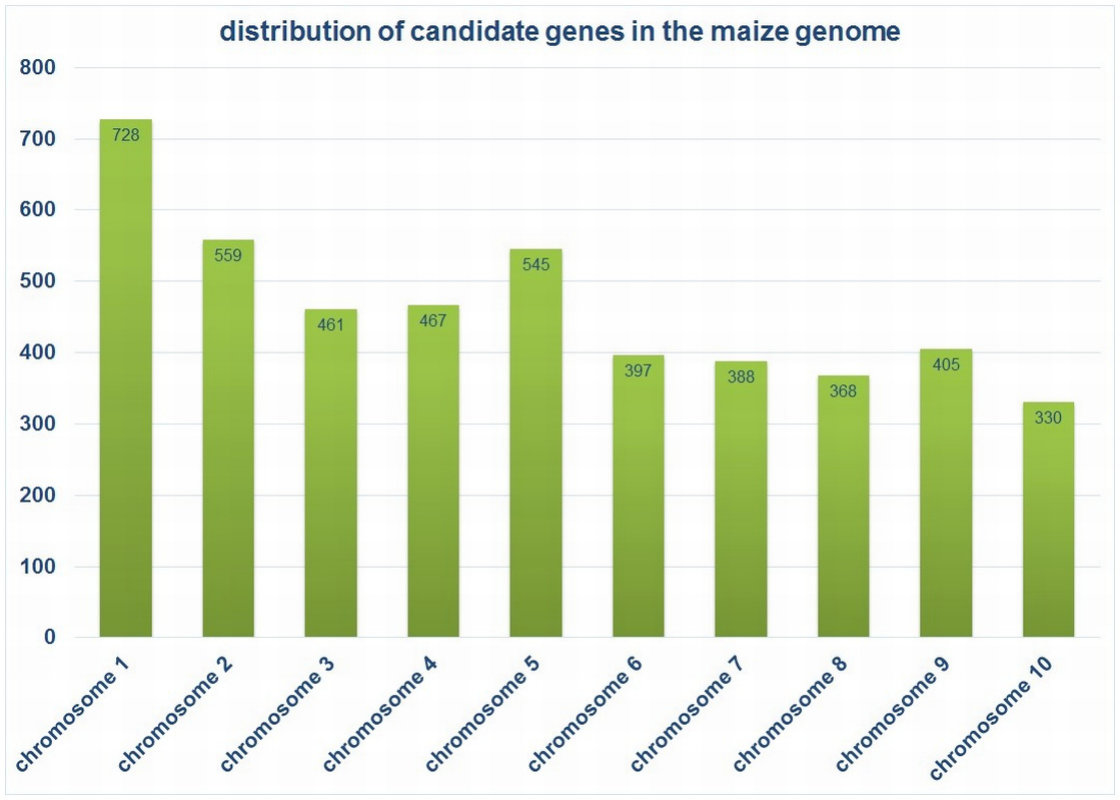
Distribution of 4,785 candidate genes over 10 maize chromosomes. Selected genes are predicted to control various aspects of plant growth, biomass production, and composition; bar graph depicts their distribution in the maize genome.

A BLAST of the candidate genes from different species (maize, rice, barley, and *Arabidopsis*) was carried out against maize CDS (maize genome project: www.maizesequence.org, version ZmB73_4a.53). Maize gene identifiers were checked for uniqueness, and genomic sequences (exons, including introns) were extracted. All genes were extended by 1 kb at the 5′ and 3′ ends, and the primary target regions to be placed on the maize array were determined. This yielded a set of sequences having an L50 of 6,583 bp, with the largest contig covering 77 kb. Using this information, and eliminating redundancy, the number of genes was reduced to 4,758 (S12 Table), corresponding to 99% of the 2.1 M NimbleGen microarray capacity. These genes were well distributed across all the maize chromosomes (Fig 7). The gene coordinates genes were then sent to Roche NimbleGen to facilitate the design of a custom 2.1 NimbleGen sequence capture microarray.

#### NimbleGen array design and algorithmic details for probe design

To avoid non-specific binding, repetitive elements were excluded from the probe design using the RepeatMasker (http://ftp.genome.washington.edu/RM/RepeatMasker.html) and WindowMasker [60] software packages. A frequency histogram of all 15mers in the target genome was constructed, and the frequencies of all 15mers forming a probe were analyzed. The average 15mer frequency was computed, and only probes having less than a 100-fold frequency were used. Uniqueness of the probes was determined by the Sequence Search and Alignment by Hashing Algorithm (SSAHA) program [61] developed at the SANGER Institute (http://www.sanger.ac.uk/resources/software/ssaha/). SSAHA probes were compared to the reference genome from which they were generated (Maize genome AGP v1, release 4a53; www.maizesequence.org). For each oligonucleotide, mapping determined if the probe matched either perfectly or closely to the genome, using a word size of 12 bp. A heuristic then accepted an oligonucleotide if its minimal match size was above the oligonucleotide length minus the word length. Therefore, if we assume an average oligonucleotide size of 50 bases, a minimal match would be expected to be at least 38 bp in length. In our design, a more stringent setting was used, allowing a minimum match size of 30. The mismatches were weighted in a trapezoidal shape, giving a higher weight to a failure in the middle of the oligonucleotide than to its end. If, in any comparison to the genome, the mismatch score was less than 10, the probe was rejected as non-unique. To use the full sequence capture array capacity, in the final probe selection replicated probes were assigned.

#### Evaluation of uniqueness settings

The design of the sequence capture array was balanced between (1) unique probes (MM1), resulting in a less optimal genome representation due to the masking of paralogous and conserved sequence domain of gene families, and (2) multiple matching probes (MMX), arrived at by the use of a less stringent probe uniqueness setting and resulting in a better coverage of the selected target genes. NimbleGen provided five independent designs with various stringency settings (MM1, MM2, MM4, MM5, and MM10). After a detailed investigation of the proposed probe sequences, we opted for MM5 (S13 Table) in order to avoid masking to many conserved gene families and/or paralogs. The consequence of lowering the uniqueness criteria is that more genes are allowed to match a given oligonucleotide, and thus specificity is reduced. However, our analysis revealed that MM5 has the optimal balance between specificity and required coverage. Using MM5, the designed Sequence Capture array covered 53.4% of target bases and 94% of our target genes, with an average probe length of 75 bp (S14 Table). This array may be ordered from Roche NimbleGen by requesting the design: 110308_ZmB73_AGP_v1_MM_cap_HX1.

A total of 4,648 target genes (97.7% of the initially targeted genes) were included in the final sequence capture array. NimbleGen utilized 2.1 million oligonucleotide probes, and if adjacent probes were in close proximity to one another they were compiled as a ‘probe cluster’. On average, each gene had 60% of its sequence covered by oligonucleotides through the use of 23,252 probe clusters.

The AGPv1 probes for the sequence capture were anchored to the maize reference (v3). The nature of the maize genome is complex, with many nearly identical paralogs. We tried to circumvent this complexity in the design of the sequence capture array via the balanced use of the multiple match parameter ‘MM5’. With our setting we successfully could limit the proportion of paralogous probes. We only observed a proportion of 2.3% of the 23,252 designed probe clustering that had an identical paralogous sequences. For further coverage analysis and estimation of the number of gaps between probes in the target region, only the uniquely anchored probes were used. Generally, probe coverage was higher in exon regions (69.2%), as compared to introns (27.0%) and the UTR regions (14.3%; S18 Table). Lower probe coverage can lead to an increase in reference positions that are not captured, literally referred to here as ‘gaps’.

We analyzed gaps between adjacent oligonucleotides in order to determine the number and sizes of gaps occurring in the target regions. We observed a well-optimized representation of target genes, especially for exons (S18 Table). Exon regions displayed fewer and shorter gaps, with only 1,801 gaps over 300 bp and 551 gaps of more than 500 bp in size. However, intron and UTR regions were characterized by a higher number of longer gaps, with more than 4,500 and 2,500 gaps in intron and UTR regions, respectively, of more than 300 bp in size (S18 Table). Our metrics show that 45% of the 26,809 total gaps are longer than 300 bp, and these large gaps are less likely to be spanned by short sequence reads. Moreover, it was expected that the number of gaps might increase when DNA from highly diverse maize lines is captured. Based on this analysis, we decided to use 454 sequencing technology, which is characterized by long sequence reads. In addition, the longer 454 reads are beneficial for correctly aligning them to the reference sequence, particularly across the highly conserved stretches of closely related genes. Consequently, 454 reads are advantageous to avoid ambiguities in the alignment of repetitive and paralogous sequence stretches (including sequence variation within these stretches).

#### Genomic DNA extraction

Fifteen seeds from each of the 21 inbred lines were planted in pots containing commercial potting soil mixtures in a greenhouse at the Institute of Plant Genetic and Crop Plant Research (IPK-Gatersleben), Gatersleben, Germany. The greenhouse has supplemental illumination using SonT Agro high pressure sodium lamp (Philips, Amsterdam, Netherlands) set to 16 h of light per day. Genomic DNA (gDNA) was isolated separately from 5 cm leaf samples of 10 randomly selected 2-week old maize seedlings from each inbred line using a modified CTAB protocol [62]. DNA was then purified by proteinase K digestion, followed by ammonium sulfate precipitation. DNA concentration and quality were assessed using a Nanodrop spectrophotometer (Willmington, DE) and electrophoresis in 0.7% (w/v) agarose gels, to verify integrity. After the normalization of DNA concentration to 250 ng/μl, equal amounts were pooled from the 10 individuals per genotype to constitute the working gDNA.

#### DNA capture

For DNA capture, 500 ng of each DNA sample was nebulized to yield fragments of approximately 250 bp to 1 kb in size. In brief, the fragmented genomic DNA samples were polished to form blunt-ended fragments, adaptors were ligated onto these fragments, and small fragments were removed. The libraries were quantified using a fluorometer, and quality was assessed using an Agilent Bioanalyzer high sensitivity DNA chip (Agilent Technologies, Santa Clara, CA, USA) to ensure libraries had a mean fragment length between 600–900 bp, with a lower size cut-off less than 10% below 350 bp, as recommended by NimbleGen protocols. Libraries that satisfied these characteristics were amplified by ligation mediated (LM)-PCR. Each sample was then evaluated with an Agilent Bioanalyzer 7500 (Agilent Technologies) to ensure a mean fragment length between 600–900 bp, as per the NimbleGen Sequence Capture protocol. The amplified sample libraries were then checked for quality to ensure the A_260_/A_280_ ratio ranged between 1.7–2.0 and sample library yield was ≥ 1.5 μg, as per NimbleGen recommendations. Subsequently, 30 μl of Plant Capture Enhancer (PCE, Roche NimbleGen, Basel, Switzerland) was added to 1.5 μg of each sample library that satisfied these characteristics, herein referred to as pre-capture amplified library, to block repetitive sequences. Hybridization buffer and hybridization component A (Roche NimbleGen) were added to each sample and loaded to a 2.1 M NimbleGen sequence capture array. The hybridization was done for 72 h at 42°C.

On completion of hybridization, each slide was washed according to NimbleGen protocols, and the bound library was eluted with 50 μl of nuclease free water. The eluted DNA was amplified by LM-PCR, and the resultant libraries, herein referred to as captured DNA, were characterized to determine concentration, size distribution, and quality. The captured DNA samples showed fragment distribution ranges from 500–1300 bp, with distribution peaks between 650 and 1000 bp.

Following the completion of the amplification reaction, samples were purified using a Qiagen QIAquick column (Qiagen, Venlo, Netherlands) following the manufacturer’s recommended protocol. The DNA was then quantified using a NanoDrop-1000 (Thermo Fisher Scientific, Waltham, Massachusetts, USA) and electrophoretically evaluated with an Agilent Bioanalyzer 7500 chip (Agilent Technologies). Aliquots of the resulting captured DNA sequencing libraries were diluted to 2 x 10^5^ molecules/μl for GS FLX sequencing.

#### Determination of captured genomic DNA enrichment

A total of 52 loci, representative of the 10 maize chromosomes, were selected from among the regions targeted by unique probes, and qPCR primer sets were designed. These were all tested together, along with one locus provided by NimbleGen (NSC-247), using the genomic DNA from different maize genotypes. Four loci that displayed good amplification across the tested genotypes were finally selected (S15 Table and S7 Fig); these are located on chromosomes 1, 3, 6, and 8.

To measure the enrichment of the captured DNA, and thus the capture efficiency, quantitative fluorescence PCR was performed on pre-captured and post-captured enriched libraries using an ABI RT PCR system. For each locus, fold enrichment = (*E*)^Δ-Ct^ was calculated, where E is the efficiency of the amplification, and Ct is the point at which the generated florescence signal rises above that of background. *E* was generated empirically for each locus using LinReg PCR analysis of Real Time PCR, and Δ-Ct was calculated by subtracting the Ct of the captured library from that of the non-captured library.

#### GS FLX DNA sequencing and raw data analysis

Captured DNA was sequenced with the GS FLX instrument at the IPK-Gatersleben (Genome Centre). The Low Molecular Weight DNA Protocol was used to prepare the 454 GS FLX sequence-ready libraries. DNA sequencing libraries for all 21 samples were prepared separately before the amplification by emPCR, following the steps described in the GS FLX emPCR Method Manual. After emPCR amplification, two prepared samples were each loaded in a half gasket PicoTiterPlate device (70_75 mm; Roche/454) and sequenced in a GS FLX system with standard Roche/454 protocols. The 454 pyrosequencing data were collected after a 7 h run on the GS FLX system, and the Roche/454 gsMapper (454 Life Sciences) was used initially to analyze all raw sequence reads that were generated.

#### Sequence analysis and alignment

The 454 reads were trimmed and filtered as described previously [29] to ensure the high data quality for all downstream analyses. To evaluate the raw read quality, clc_quality_trim (CLC assembly cell, version 4.2) was used to trim reads that did not achieve sufficient base pair quality standards (minimal quality PHRED score >20), excluding short sequences (<30 bp).

#### Maize genome references

Sequences were aligned to the B73 reference genome (AGPv3.20) using various read alignment programs. The genomic reference and corresponding gene models (GFF, cDNA, and protein sequence) were downloaded from Gramene [63] (http://ensembl.gramene.org/Zea_mays/Info/Index).

#### Conversion of target regions to maize reference v3 (AGPv3.20)

The design of the capture array was based on the maize reference AGPv1. However, all mappings and further downstream analysis were performed on the AGPv3.20 reference. To anchor AGPv1 based MM5 positions to the AGPv3.20 maize reference, two approaches were applied. For target genes with an identifier present in both reference versions, their corresponding gene positions were used. For the 306 genes with no corresponding gene identifier in v3 reference, these target regions were anchored to the AGPv3.20 reference by performing BLASTN [64] analyses with an identity threshold of 98%, using BLAST version 2.2.28. Using these two approaches, all MM5 target gene positions were anchored to AGv3.20 reference (S13 Table).

#### *De novo* assembly

A *de novo* assembly of the quality trimmed 454 reads was performed using Newbler (version 2.6, 454 Life Sciences) with the default settings. We also re-sequenced B73 for our targeted regions with the 454 platform and mapped it to the respective maize reference genome. This was used as a control to identify regions of the B73 assembly that were captured by our designed array. The resulting genotype specific assemblies were analyzed with BLASTN (identity 99.8) against the selected target gene reference models (cDNA) to estimate the presence of a gene. A threshold of 75% gene sequence length absence relative to the parent gene sequence length in reference was used to as model to define absence of a gene in a given inbred line.

#### Read alignment evaluation

Seven read alignment tools (BWA-SW, BWA-MEM, Bowtie2, CLC mapper, Smalt, Stampy, and NextGenMap) were used to evaluate read alignment methods. This collection of alignment tools consists of widely used standard tools, as well as recently published tools that are able to handle Illumina and 454 reads. For BWA, the previous version, BWA-SW, for long reads, and a recently released version, BWA-MEM, which also is suitable for long reads, were used. Stampy is the preferred read aligner for Illumina reads but performed well in our study in handling 454 reads. CLC mapper is a commercial tool released within the CLC assembly cell repository. An in-depth evaluation of read alignment tools was performed on a large-scale utilizing all 21 genotype lines, in three independent parameter configurations. Besides the standard parameter settings, we utilized for each read aligner, two additional custom settings aiming for a higher sensitivity. The detailed settings are provided in S2 File. To compare mapping results, the GATK [46] tool repository was used. Utilizing the combined reads of all 21 inbred lines, regions with sufficient coverage (—cov 20) were screened and then extracted with ‘FindCoveredIntervals’. Coverage in target regions was discovered with ‘DepthOfCoverage’ using our target coordinates (S4 Table). Genes absent in certain inbred lines were detected by applying BEDtools 'coverage' software [65] to the individual mappings (BWA-MEM) per inbred line.

#### Variant detection

A wide range of read alignment and SNP calling tools was used to establish the most favorable set of SNPs in our maize panel, and VCFtools [66] was utilized to calculate statistics for each SNP data set. A high number of INDELs is expected, since 454 reads have an inherently higher error rate at long homopolymers stretches [67], which can lead to false positive INDEL detection. To avoid this, the maize genome was screened for homopolymers equal to, or larger than, 7 bp, and the detected INDELs that were embedded, or adjacent to, homopolymers were classified as false positives and discarded. Additional criteria, such as that a basic INDEL position must be supported by at least two variant callers, were applied to further filter the INDEL positions. Two approaches can be utilized to filter VPs: the first is an internal filtering, with parameter settings that aim to reveal a more stringent variant calling. In addition, subsequent to the variant calling process, predicted candidates can be analyzed and filtered. This *posterior* filtering plays an important role in variant discovery and has a crucial impact on reaching a high quality prediction [68]. However, in our study we tried to estimate the sensitivity of a variant calling method without the influence of a user defined parameter setting and therefore compared the unbiased default settings. In addition, an in-depth evaluation of variant calling methods was performed, utilizing a randomly selected inbred line (‘NC358’). For this selected line, we utilized all 21 pre-calculated alignments for subsequent variant calling. Besides the standard parameter settings, we used two additional custom settings for each variant calling method, aiming for a higher sensitivity, and totaling to 504 tested combinations. The detailed settings are provided in S2 File.

#### Diversity assessment

To measure the detection power and correctness of each evaluated variant caller, we calculated sensitivity, specificity, and F_1_-score, as shown below:

$$Sensitivity\;Se\;=\;\frac{number\;of\;true\;positive\;sites}{condition\;positive\;sites}\;=\;\frac{TP}{\left(TP+FN\right)}$$

$$Specificity\;Sp\;=\;\frac{number\;of\;true\;negative\;sites}{condition\;not\;polymorph\;sites}\;=\;\frac{TN}{\left(TN+FP\right)}$$

$$F_{1}score\;F_{1}{=}_{\;}\frac{\left(2\;*TP\right)}{{\left((2*TP)+\;FN+FP\right)}_{\;}}$$

The sensitivity measure indicates the power of a tool to detect true positive sites (designated TP in the equations above). The specificity estimates the true negative (designated TN in the above equations) detection rate, indicating the ability of a particular variant calling tool to discard true negative calls. The F_1_-score is the harmonic mean, balancing precision and sensitivity. Four control data sets were investigated to evaluate and validate variant predictions: 1) the genotyping array (50k) comprising of 52,374 SNP markers [5], 2) the genotyping-by-sequencing (GBS) comprising of 719,487 [39], 3) the RNA-Sequencing (RNAseq) comprising of 931,484 markers within the gene space of maize [40], and 4) the large data set of the maize HapMap2 (HapMap2) comprising of over 55 million SNPs (http://data.iplantcollaborative.org/quickshare/e75bc315fc0f9fda/HapMapV2RefgenV220120328.vcf.gz) [41].

A *‘true positive site’* refers to a variant position that was predicted in our data set and is matching a position in the corresponding control data set. The intersection of a particular control dataset and the detected VPs that are validated by at least one of the evaluated variant calling methods or settings defines the *‘condition positive sites’* (CP) represented in our data (Table 3). Subsequently, these CP are used to classify a prediction of a particular variant calling method as *‘true positive’* or *‘false positive’* (FP). As described above, the F_1_-score is defined as the harmonic mean between sensitivity and precision, where the latter estimates the ratio between TP and FP. For the F_1_-score calculation, we extended this set to include all VPs that have an overlap to our final ‘CornFed Target Diversity’ (CTD) dataset. This strategy aims to circumvent the severe underestimation of true positive sites that would occur if only the less pronounced overlap to the control dataset was classified as TP, where all other predictions would be automatically discarded and judged as FP. Finally, the set of *‘false positive’* sites is defined as the opposite of the TP, meaning that a FP site is not in the control dataset and not in our CTD set. FP sites refer to VP candidates that do not have enough support by independent variant calling, as defined by the CTD setting (three independent variant calling methods). We then define all positions that have on average a minimal coverage of one read, but were not called as polymorphs, as *‘true negative’* (TN) sites. In conjunction with sites classified as FP, these TN sites define the complete set of ‘*condition not polymorph sites’ (CNP)*. To evaluate the prediction power and the optimal detection method, we assessed each of the eight variant calling tools and analyzed their predictions for each of the 21 maize inbred lines. This evaluation has been extended for one genotype (NC358) by using multiple parameter settings (S2 File). To assess the overlapping and non-overlapping VPs among the four control and CTD data sets we constructed a diagram for illustration. The Venn diagram was created using the web tool provided by the Bioinformatics and Systems Biology of Gent (http://bioinformatics.psb.ugent.be/webtools/Venn).

#### Genotype concordance

All the 21 maize inbred lines re-sequenced at specific target genes were also genotyped with the 50k maize genotyping array [5] (S2 Table). Individual variant discovery was also performed for each genotype with the eight variant callers. Subsequently, considering our target genomic regions that were also genotyped with 50k array, the overlap between the 50k genotyping and that of the variant callers was computed, and the called alleles were analyzed for genotype concordance. Eighteen of the 21 inbred lines (excluding Lo11 and Mo24W in which the analysis failed, and B73 which is the reference genome) were used in this analysis.

#### Combinatorial variant calling approach to detect final SNPs

The final SNP set was established by posterior filtering. Respective positions fulfilled the following criteria: 1) VP has a minimal read coverage of five, 2) VP is in bi-allelic sites, 3) VP exceeds the minimal quality score (>0.4 normalized quality score), and 4) VP is predicted with at least three independent variant calling methods. This cross validation parameter of three tools was investigated to verify a prediction and to achieve an optimal result within our heterogeneous maize collection. The threshold of three independent tools supporting a prediction was defined to be the optimal trade-off between sensitivity and specificity. Therefore we validated various thresholds (1–8) and their combined prediction power using the results of the 50k genotyping data set of all 21 inbred lines. To calculate the F_1_-score false negative and false positive sites had to be considered. FN sites are VPs that were missed from the 50k array (proportion with overlap to our target sequencing) and missed from the CTD set. FP sites are positions that were predicted but have no overlap to any of these two control sets.

#### SNP annotation

The final SNP set was annotated using the software COOVAR [69].

#### Genetic relationship between 21 inbred maize lines

Phylogenetic clustering of the final SNP data set was performed with SNPhylo [70] with standard settings. A graphical representation was generated with Circos [71] using the final set of SNPs. A total of approximately 1 million variants detected in all 21 inbred lines were used to generate a graphic presentation. For clarity of presentation, SNP densities were displayed in regions of 500 kbp (window size).

#### Population structure

The software STRUCTURE [72,73] was used to analyze the population structure based on the admixture model, where each individual draws some fraction of its genome from each of the K populations. This method is useful to identify population stratification, since inbred lines whose genotypes indicate admixture are assigned jointly to two or more populations. The correlated allele frequencies model [74] which often improves clustering for closely related populations was used. Ten runs of STRUCTURE were carried out for each set of K sub-populations, with K values from 2 to 10. The choice of the number of K (2–10) was based on earlier cluster analysis using SNP profile, which gave an estimate of 7–8 grouping of the studied inbred lines. The ad hoc [75] criterion was used to determine the optimum value of K.

#### Presence and absence of genes

To assess the absence of genes in the studied inbred lines, we used BEDtools [65]. The coverage of captured genes was computed from all BWA-MEM generated read alignments (BAM files). To discover the sequence captured for each gene, we first analyzed the presence of target gene reads in the re-sequenced B73 454 sequence reads. Subsequently, the presence of the represented proportion was analyzed in all other re-sequenced maize inbred lines. Minimal read depth threshold was set to 2, and the covered gene length was analyzed with different thresholds (minimal gene sequence length 0%, 10%, 20%, and 25%) to determine if a given gene is present in, or absent from, a given inbred line. A gene was declared present in any given inbred line if more than 25% of its sequence length (according to the re-sequenced B73) was covered by reads; otherwise, it was declared absent. Complete target gene sequence length coverage in the B73 reference was determined using two thresholds, i.e., 80% and 90%. To further validate the absence of a gene, a BLASTN [64] analysis was performed on the *de novo* assembly of a given genotype for a given gene declared as absent.

#### Functional annotation of target genes

Functional annotation of the target genes was carried out using BLAST2GO [76]. The analysis of the three ontology classes (biological processes, molecular function, and cellular component) was performed for all the target genes. The third level of the GO hierarchy was used to subdivide the gene set into clusters (S16 Table and S8 Fig). We then analyzed the resulting categorization for presence-absence genes and for presence of radical mutation on the target genes. In addition, for the final candidate genes, pathway and a further enzymatic annotation from MaizeCyc [77] information were integrated.

#### Accession numbers

The raw 454 DNA sequence data obtained from sequence capture and re-sequencing of the 21 maize inbred lines have been deposited at the European Nucleotide Archive (ENA) in the Sequence Read Archive (SRA) and are available under the following EBI project ID: PRJEB5496, [http://www.ebi.ac.uk/ena/data/view/PRJEB5496]. Details are provided in S17 Table.

## Supporting Information

S1 Fig In-depth evaluation of read alignment methods, utilizing three different parameter settings. For each particular setting, the mapping is evaluated for all 21 inbred lines by determining the number of aligned reads, the number of aligned read on target, the mutual agreement of the alignment (‘number of reads mapped by other method’), the uniquely aligned reads, and the number of reads aligned by the majority of methods. The graphic shows the results for genotype NC358, which was selected as example.
(TIF)

S2 Fig Heat map showing the impact of read alignment (seven read alignment methods) on diversity detection (eight variant detection methods). The analysis was performed on the random selected genotype ‘NC358’, and all 504 possible combinations of alignment and variant calling methods (three parameter settings) were included. To construct the heat map, the detected VPs of each individual approach were compared to the 50k data to reveal true positive predictions.
(TIF)

S3 Fig Application of different cut-off values for the variant caller count (VCC). All VPs in our studied lines that overlap a 50k position are considered true positive. The proportion of the total number of true positives is depicted in blue for each VCC value. The F_1_-score, shown in yellow, illustrates the impact of false positive and false negative values. The harmonic mean reaches highest values at VCC3.
(TIF)

S4 Fig STRUCTURE analysis to estimate the value of K for optimal partitioning of data. (TIF)

S5 Fig Genotype independent distribution of diversity density, shown for the 10 maize chromosomes. Chromosome bins with a size of 2 Mbp were analyzed, and regions that are characterized with high diversity in our genotype collection are indicated in yellow.
(TIF)

S6 Fig Circos histogram plot for the captured target gene diversity in 21 maize inbred lines. Chromosomes were analyzed in 500 kbp bins, and the genotypes are shown in the identical order as depicted in Fig 6.
(TIF)

S7 Fig DNA gel electrophoresis for qPCR control loci. (JPG)

S8 Fig Pie chart of Blast2GO annotations of biological processes, molecular function, and cellular components within the set of target genes. The Blast2GO hierarchy is presented at level three for all three categories.
(TIF)

S1 Table Measurement of B73 sequence enrichment using qPCR. (XLSX)

S2 Table Genotyping of 21 maize inbred lines using 50k Illumina array. (XLSX)

S3 Table Statistics of *de novo* assembly for 21 maize inbred lines. (XLSX)

S4 Table SNP annotation for all 21 maize inbred lines. (XLSX)

S5 Table Survey of the SNPs in coding regions of the re-sequenced genes in each of the inbred lines. The number of non-synonymous coding SNPs and nonsense SNPs, and the number of genes they affect is also shown.
(XLSX)

S6 Table List of genes that are affected by a SNP mutation leading to radical protein changes for each genotype. (XLSX)

S7 Table Geographic origin, including pedigree data, of genotypes, and a short description of maize inbred lines. (XLS)

S8 Table Joint analysis of phenotypic information (high and low biomass) in combination with existence of a radical mutation identified in candidate genes. (XLSX)

S9 Table List of captured target genes providing a comprehensive overview of the capture array sequencing. The resulting sequence coverage is presented as percentage of gene length.
(XLSX)

S10 Table List of captured target genes, including the assigned biological function. A joint analysis links the phenotypic information of lowest yielding (B111 and EA1070) and highest yielding (F2 and F7) inbred lines with the presence and absence information. A gene was declared absent if at least 75% of sequence length is missing, and the captured gene length of maize inbred B73 is used as reference.
(XLSX)

S11 Table Inventory of candidate genes for the NimbleGenArray design, including a description of target genes predicted to be involved in the modification of biomass accumulation and production, as well as in water use. (XLS)

S12 Table Candidate genes selected for designing the 2.1 M NimbleGen sequence capture microarray. (XLS)

S13 Table NimbleGen array design providing positions of selected target genes anchored in AGPv1 and AGPv3. (XLSX)

S14 Table Array design statistics. (XLS)

S15 Table Forward and reverse primer sequences for four different IPK control loci. (XLSX)

S16 Table Blast2GO annotation for biological processes, molecular function, and cellular components identified within the set of target genes. (XLSX)

S17 Table EBI submission details of the raw data. (XLSX)

S18 Table Extended analysis of the NimbleGenArray design. The analysis included 4,342 non-overlapping genes that were mapped uniquely to the maize genome reference (v3). Results revealed different representations of sequence capture probes in UTR, exonic and intronic regions of studied genes.
(XLSX)

S1 File List of 383,145 variant positions, including SNP annotation and PIC information. (TXT)

S2 File List of parameter settings that have been applied for each of the integrated read alignment and variant calling method. (TXT)
