# Supplementary figures and images for: Targeted Sequencing Reveals Large-Scale Sequence Polymorphism in Maize Candidate Genes for Biomass Production and Composition
Source: PLoS One. 2015 Jul 7;10(7):e0132120. doi: 10.1371/journal.pone.0132120 (PMC4495061; doi:10.1371/journal.pone.0132120)

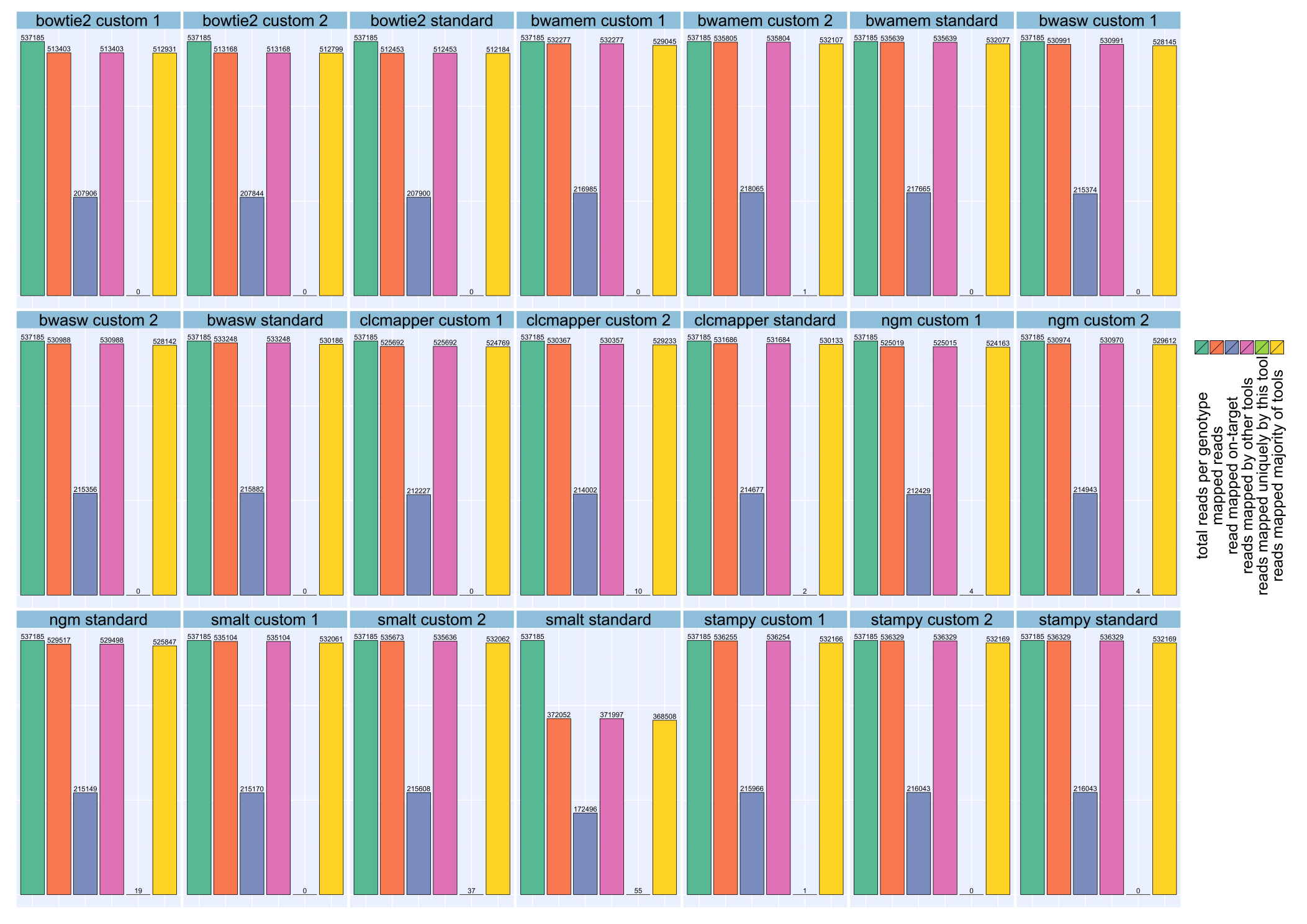

Supplement: S1 Fig — For each particular setting, the mapping is evaluated for all 21 inbred lines by determining the number of aligned reads, the number of aligned read on target, the mutual agreement of the alignment (‘number of reads mapped by other method’), the uniquely aligned reads, and the number of reads aligned by the majority of methods. The graphic shows the results for genotype NC358, which was selected as example. (TIF) [file pone.0132120.s001.tif]

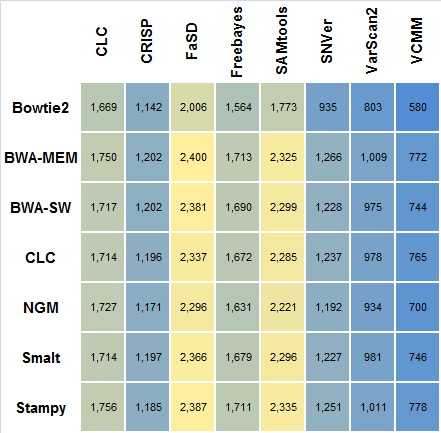

Supplement: S2 Fig — The analysis was performed on the random selected genotype ‘NC358’, and all 504 possible combinations of alignment and variant calling methods (three parameter settings) were included. To construct the heat map, the detected VPs of each individual approach were compared to the 50k data to reveal true positive predictions. (TIF) [file pone.0132120.s002.tif]

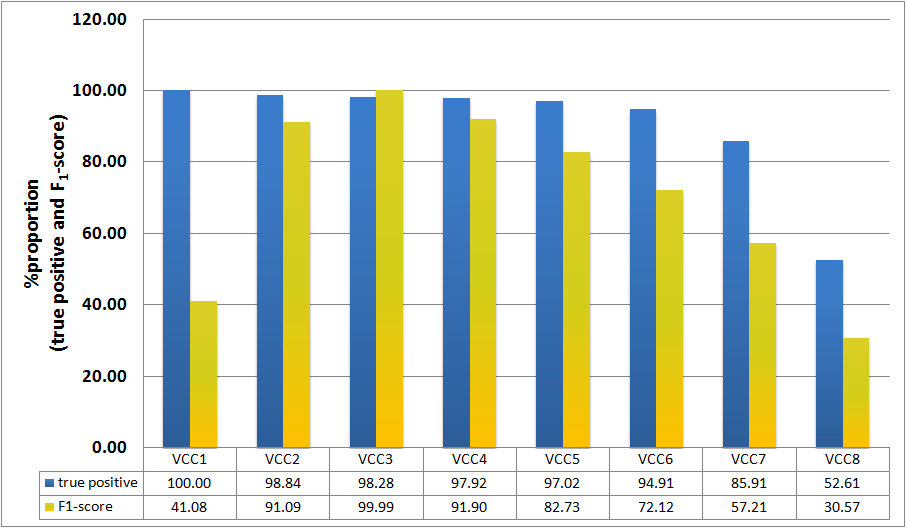

Supplement: S3 Fig — All VPs in our studied lines that overlap a 50k position are considered true positive. The proportion of the total number of true positives is depicted in blue for each VCC value. The F1-score, shown in yellow, illustrates the impact of false positive and false negative values. The harmonic mean reaches highest values at VCC3. (TIF) [file pone.0132120.s003.tif]

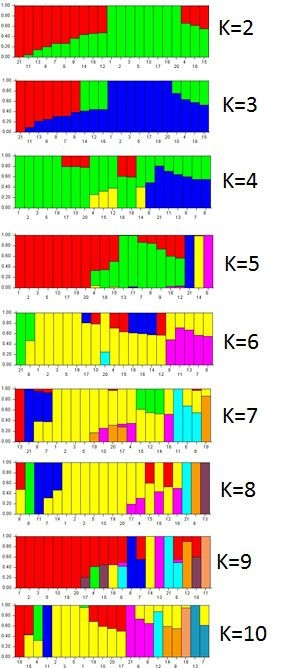

Supplement: S4 Fig — (TIF) [file pone.0132120.s004.tif]

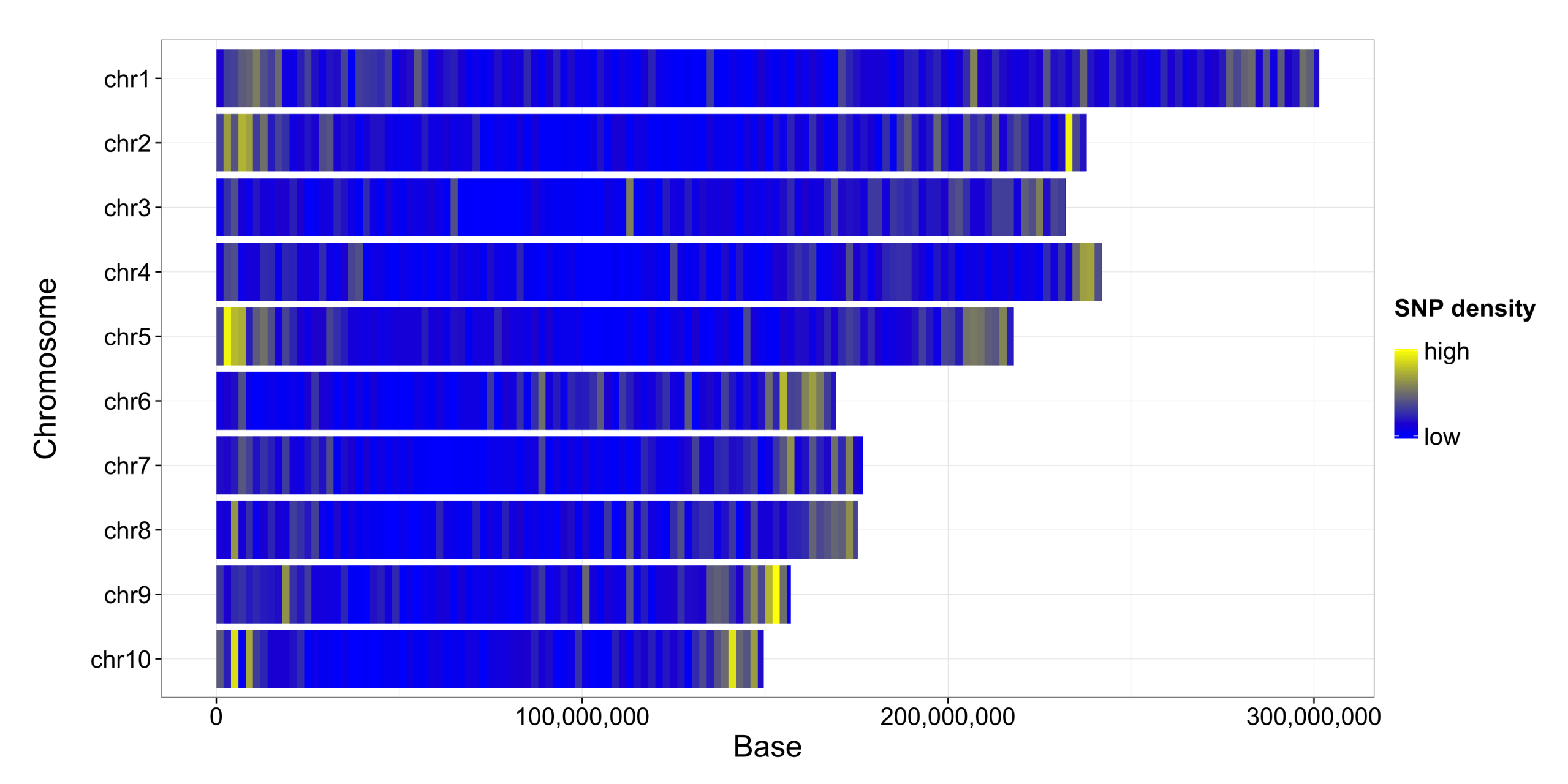

Supplement: S5 Fig — Chromosome bins with a size of 2 Mbp were analyzed, and regions that are characterized with high diversity in our genotype collection are indicated in yellow. (TIF) [file pone.0132120.s005.tif]

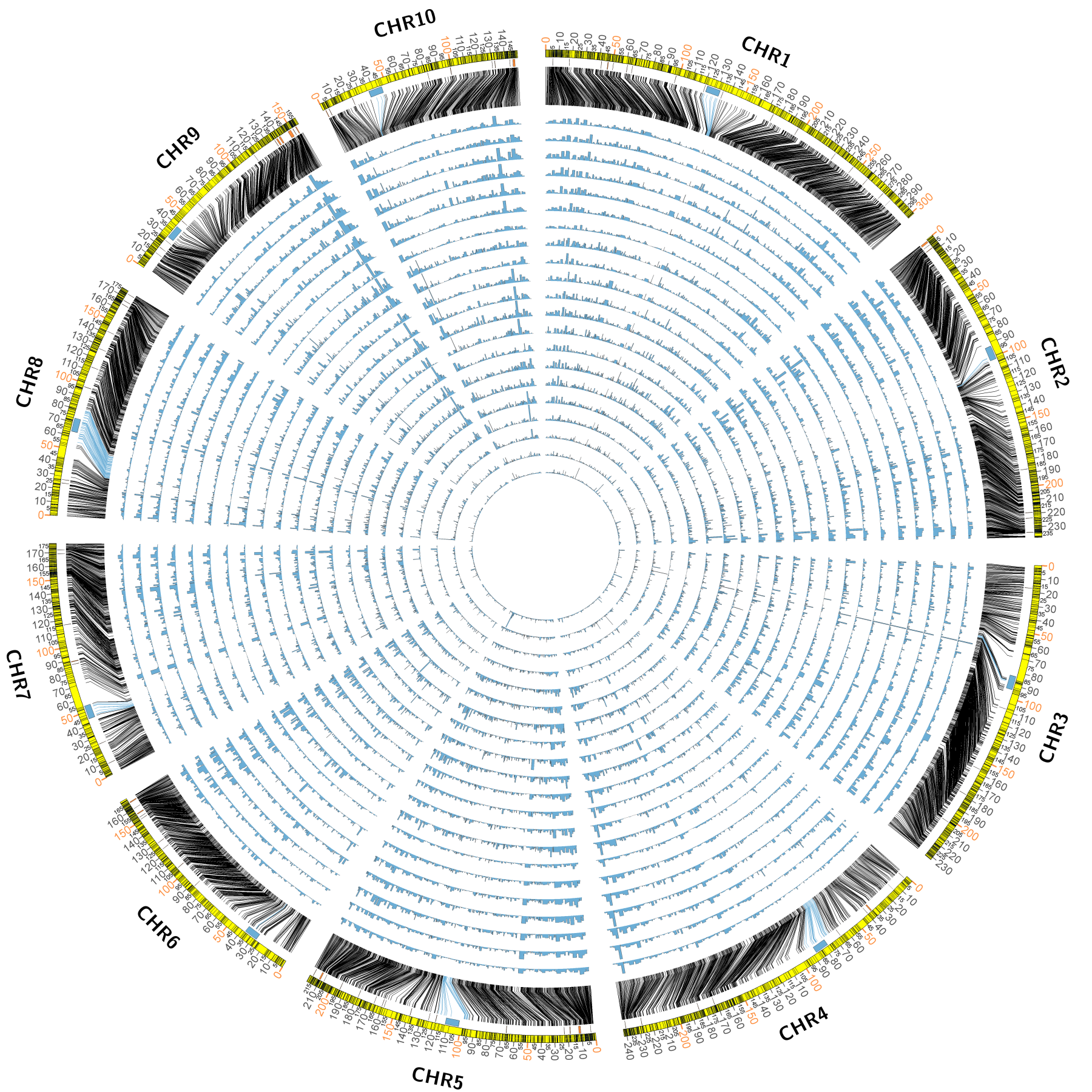

Supplement: S6 Fig — Chromosomes were analyzed in 500 kbp bins, and the genotypes are shown in the identical order as depicted in Fig 6. (TIF) [file pone.0132120.s006.tif]

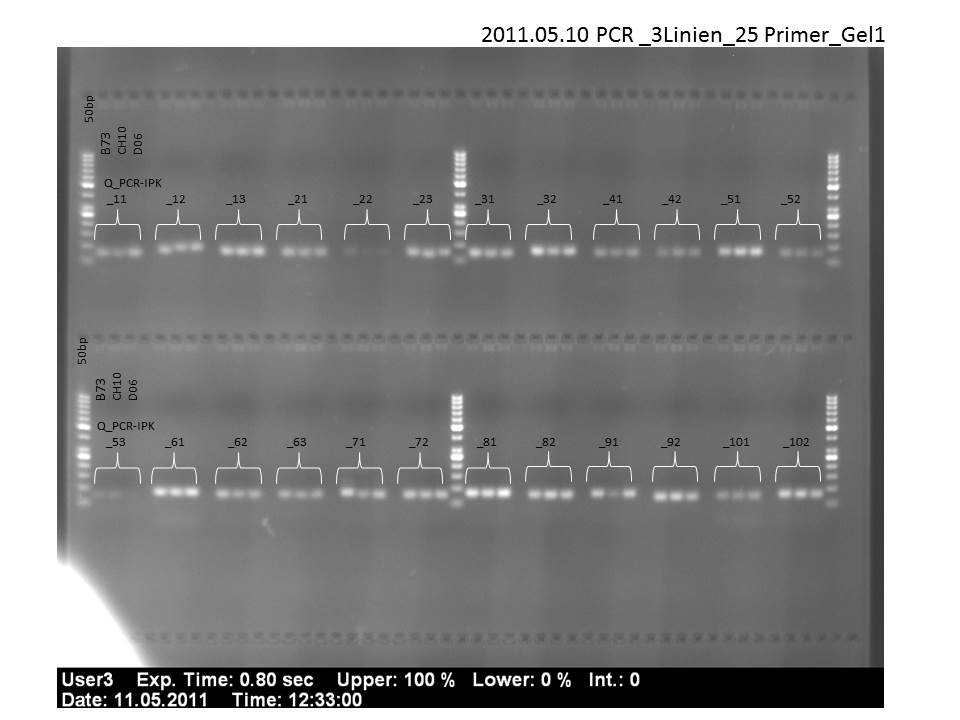

Supplement: S7 Fig — (JPG) [file pone.0132120.s007.jpg]

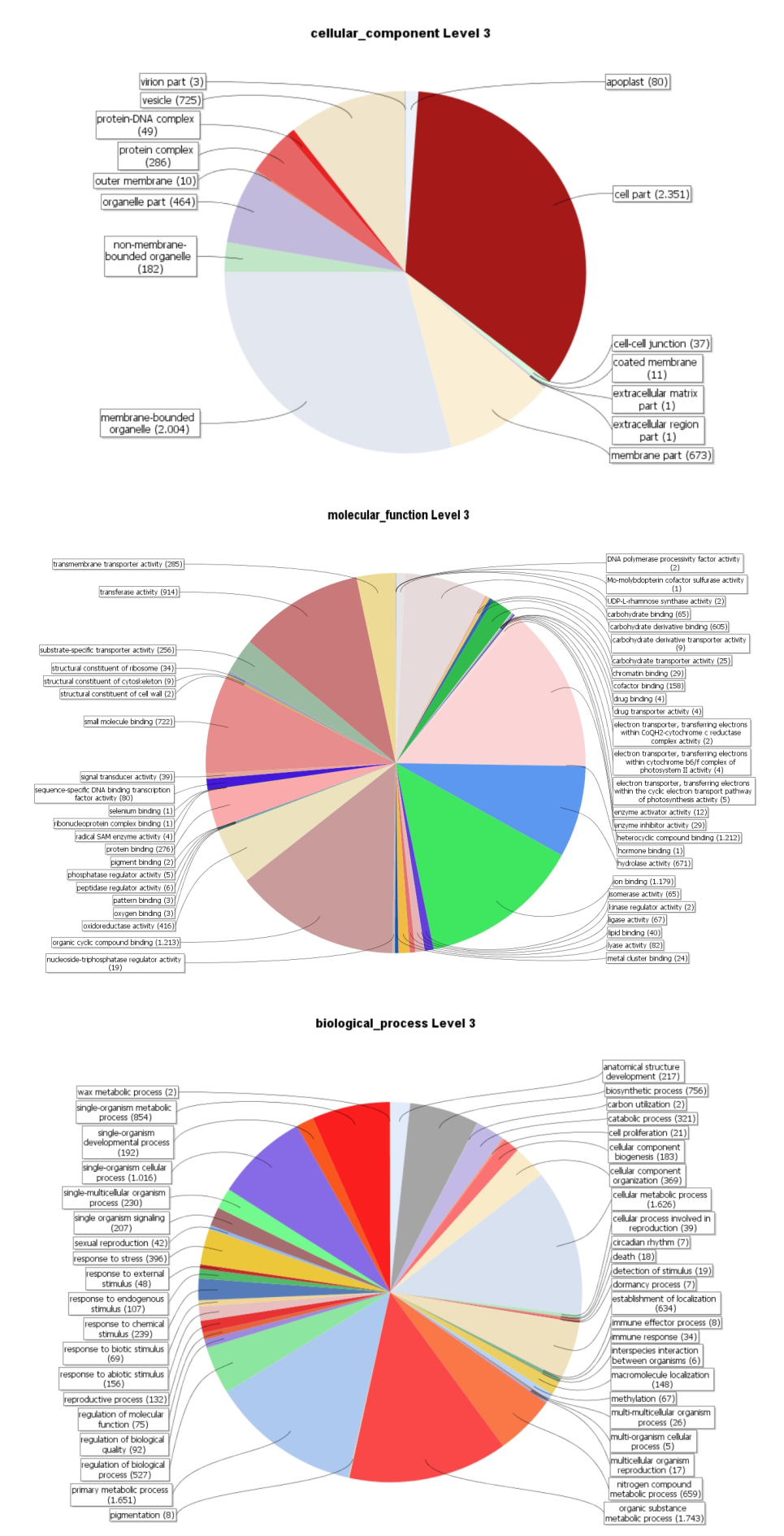

Supplement: S8 Fig — The Blast2GO hierarchy is presented at level three for all three categories. (TIF) [file pone.0132120.s008.tif]
